# The PI3K-AKT-mTOR Pathway and Prostate Cancer: At the Crossroads of AR, MAPK, and WNT Signaling

**DOI:** 10.3390/ijms21124507

**Published:** 2020-06-25

**Authors:** Boris Y. Shorning, Manisha S. Dass, Matthew J. Smalley, Helen B. Pearson

**Affiliations:** The European Cancer Stem Cell Research Institute, Cardiff University, Hadyn Ellis Building, Maindy Road, Cardiff CF24 4HQ, Wales, UK; ShorningB@cardiff.ac.uk (B.Y.S.); DassMS@cardiff.ac.uk (M.S.D.); SmalleyMJ@cardiff.ac.uk (M.J.S.)

**Keywords:** AKT, AR, castration-resistant prostate cancer (CRPC), MAPK, mTOR, PI3K, prostate cancer, therapeutic resistance, WNT

## Abstract

Oncogenic activation of the phosphatidylinositol-3-kinase (PI3K), protein kinase B (PKB/AKT), and mammalian target of rapamycin (mTOR) pathway is a frequent event in prostate cancer that facilitates tumor formation, disease progression and therapeutic resistance. Recent discoveries indicate that the complex crosstalk between the PI3K-AKT-mTOR pathway and multiple interacting cell signaling cascades can further promote prostate cancer progression and influence the sensitivity of prostate cancer cells to PI3K-AKT-mTOR-targeted therapies being explored in the clinic, as well as standard treatment approaches such as androgen-deprivation therapy (ADT). However, the full extent of the PI3K-AKT-mTOR signaling network during prostate tumorigenesis, invasive progression and disease recurrence remains to be determined. In this review, we outline the emerging diversity of the genetic alterations that lead to activated PI3K-AKT-mTOR signaling in prostate cancer, and discuss new mechanistic insights into the interplay between the PI3K-AKT-mTOR pathway and several key interacting oncogenic signaling cascades that can cooperate to facilitate prostate cancer growth and drug-resistance, specifically the androgen receptor (AR), mitogen-activated protein kinase (MAPK), and WNT signaling cascades. Ultimately, deepening our understanding of the broader PI3K-AKT-mTOR signaling network is crucial to aid patient stratification for PI3K-AKT-mTOR pathway-directed therapies, and to discover new therapeutic approaches for prostate cancer that improve patient outcome.

## 1. Introduction

Prostate cancer is the second leading cause of cancer-related deaths in men worldwide, despite extensive efforts to raise awareness and significant advancements in detection, screening, and treatment approaches [1,2,3]. Although patients with localized prostate cancer generally have a good prognosis, the 5-year relative survival rate is significantly reduced for patients that present with metastatic prostate cancer at diagnosis [4]. ADT and/or radiotherapy remains the mainstay treatment for patients that relapse post-surgery. ADT involves blocking the production of androgen in the testes via the hypothalamus-pituitary-gonadal axis with luteinizing hormone releasing hormone (LHRH) agonists (e.g., Leuprolide) or antagonists (e.g., Degorelix). Although prostate tumors respond initially to ADT, the emergence of androgen-independent, castration-resistant prostate cancer (CRPC) invariably occurs and the outcome is poor [5,6,7,8]. Treatment options for CRPC and patients with metastatic disease at diagnosis include chemotherapy, radium-223, second generation anti-androgens (e.g., the Cytochrome P450 17A1 (CYP17A1) inhibitor abiraterone acetate that prevents androgen biosynthesis, or enzalutamide that targets AR directly), and clinical trials [5,6,8,9,10]. However, CRPC remains incurable and new biomarkers and treatments for prostate cancer and CRPC are in high demand.

**Figure 1:**
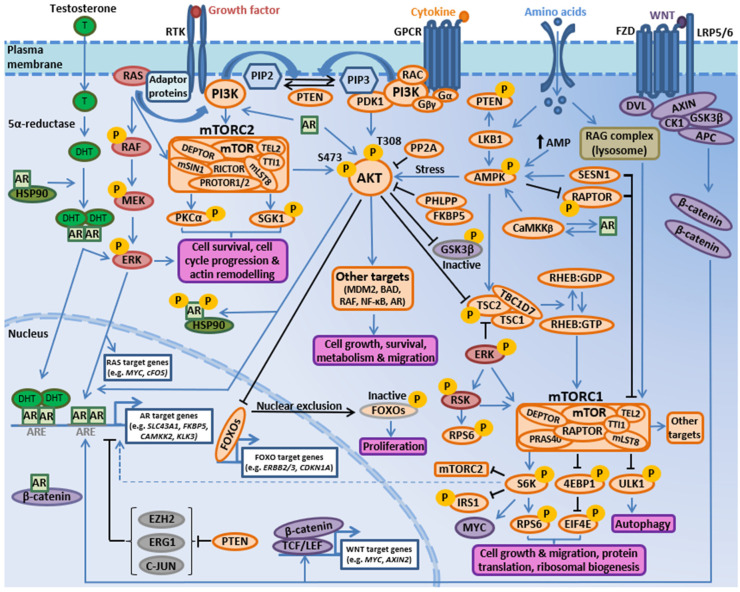
PI3K-AKT-mTOR signaling interaction with the AR, MAPK, and WNT pathways. Image displays a model of PI3K-AKT-mTOR signaling via Class IA PI3Ks, and crosstalk with AR, RAS/MAPK, and WNT signaling cascades. 4EBP1, eukaryotic initiation factor 4E binding protein 1; AMP, adenosine monophosphate; AMPK, 5′ AMP-activated protein kinase; APC, adenomatous polyposis coli; ARE, androgen responsive element; AXIN, axis inhibition protein; BAD, Bcl-2-associated death promoter; c-JUN, transcription factor AP−1; CaMKKβ, Ca(2+)/calmodulin-dependent protein kinase kinase β; CK1, casein kinase 1; DEPTOR, DEP domain-containing mTOR-interacting protein; DHT, dihydrotestosterone; DVL, dishevelled; EIF4E, eukaryotic translation initiation factor 4E; *ERBB2/3*, Erb-B2 receptor tyrosine kinase 2, that encodes human epidermal growth factor 2/3 (HER2/3); ERG1, ETS-related gene 1; ERK, mitogen-activated protein kinase 1/3; EZH2, enhancer of zeste homolog 2; *FKBP5*, FK506 binding protein 5; FOXO, forkhead box protein O; FZD, frizzled family receptor; GDP, guanosine diphosphate; GPCR, G-protein coupled receptor; GSK3β, glycogen synthase kinase 3 beta; GTP, guanosine triphosphate; HSP90, heat-shock protein 90; IRS, insulin receptor substrate; *KLK3*, kallikrein related peptidase 3 (encoding prostate specific antigen, PSA); LKB1, liver kinase B1; LRP5/6, low-density lipoprotein receptor-related proteins 5 and 6; LEF, lymphoid enhancer binding factor 1; MAPK, mitogen-activated protein kinase; MDM2, mouse double minute 2 homolog; MEK, mitogen-activated protein kinase kinase; mLST8, mTOR associated protein LST8 homolog; mSIN1, mitogen-activated protein kinase associated protein 1 (MAPKAP1); mTOR, mammalian target of rapamycin; mTORC1/2, mTOR complex 1/2; NF-κB, nuclear factor kappa light chain enhancer of activated B cells; P, phosphorylation event; PDK1, phosphoinositide dependent kinase 1; PHLPP, PH domain leucine-rich repeat protein phosphatase; PKCα, protein kinase C alpha; PP2A, protein phosphatase 2A; PRAS40, proline-rich AKT substrate of 40 kDa; PROTOR1, protein observed with Rictor-1; PROTOR2, protein observed with Rictor-2; RAF, rapidly accelerated fibrosarcoma; RAG, recombination activating genes; RAPTOR, regulatory-associated protein of mTOR; RHEB, RAS homolog enriched in brain; RICTOR, rapamycin-insensitive companion of mTOR; RPS6, ribosomal protein S6; RSK, 90 kDa ribosomal S6 kinase; RTK, receptor tyrosine kinase; S6K, p70 ribosomal S6 kinase; SESN1, sestrin 1; SGK1, serum/glucocorticoid-regulated kinase 1; *SLC43A1*, solute carrier family 43 member 1 (encoding l-type amino acid transporter 3, LAT3); T, testosterone; TBC1D7, Tre2-Bub2-Cdc16 domain family member 7; TCF, T cell factor; TEL2, telomere length regulation protein (or telomere maintenance 2, TELO2); TSC1, Tuberous sclerosis complex 1; TCS2, tuberous sclerosis complex 2; TTI1, TELO2 interacting protein 1; ULK1, Unc-51 like autophagy activating kinase 1; WNT, WNT ligand. Figure based on previous work [12,14,19,20,21,22,24,25].

PI3K-AKT-mTOR signaling is elevated in a high proportion of prostate cancer patients, and CRPC is associated with increased activation of the PI3K-AKT-mTOR pathway [11,12,13]. Accordingly, PI3K-AKT-mTOR pathway inhibitors are currently being explored as therapeutic agents against hormone-sensitive prostate cancer and CRPC [11,12,13,14,15,16,17]. PI3Ks are a large family of lipid kinase enzymes divided into three classes termed Class I (subdivided into Class IA and IB), Class II, and Class III, reflecting substrate specificity and subunit organization [18,19,20]. Class IA PI3Ks are heterodimers containing a catalytic subunit (p110α, p110β, or p110δ, encoded by *PIK3CA*, *PIK3CB* and *PIK3CD* respectively) and a regulatory subunit (p85α/p55α/p50α, p85β or p55γ, encoded by *PIK3R1*, *PIK3R2* and *PIK3R3* respectively) that controls protein localization, receptor binding, and activation [19,20,21]. Class IA isoforms are ubiquitously expressed, except for p110δ and p55γ that are primarily expressed in the hematopoietic/central nervous systems and testes [19,20,21,22]. Receptor tyrosine kinases (RTKs) can activate p110α, p110β, and p110δ catalytic isoforms, whereas the p110β isoform can be additionally activated by G protein-coupled receptors (GPCRs) [19,20,21,22] (Figure 1). The small GTPase RAS can also directly activate p110α and p110δ, while Rho-GTPases (e.g., RAC) are reported to activate p110β [20]. Once activated, Class IA PI3Ks initiate a wave of downstream signaling events by synthesizing the lipid secondary messenger phosphatidylinositol 3, 4, 5 trisphosphate (PIP3) from phosphatidylinositol 4,5 bisphosphate (PIP2) to mediate cell growth, proliferation, autophagy, and apoptosis [19,21]. The tumor suppressor, phosphatase and tensin homolog deleted on chromosome 10 (PTEN), negatively regulates PI3K-AKT-mTOR signaling by converting PIP3 back to PIP2 [23] (Figure 1).

Elevated PIP3 levels lead to the activation of multiple kinases, including PDK1, which phosphorylates downstream targets such as AKT at residue Thr308 [19,21,26,27,28]. Activated AKT phosphorylates numerous substrates to regulate vital cellular processes, including FOXOs, GSK3β, NF-κB, and TSC2 [19,21,26,27,28]. For instance, TSC2 phosphorylation by AKT inactivates RHEB, which potentiates mTORC1 signaling and results in the inhibition of autophagy and increases cell growth, protein translation and ribosomal biogenesis via the subsequent phosphorylation of mTORC1 substrates such as ULK1, S6K, and 4EBP1 [27,29]. Phospho-S6K can also phosphorylate RICTOR to regulate mTORC2 signaling [30]. mTORC2 phosphorylates multiple downstream targets to mediate cell survival, cell cycle progression, and actin remodeling. These include AKT at residue Ser473, which leads to AKT hyperactivation, serum/glucocorticoid-regulated kinase 1 (SGK1) and protein kinase Cα (PKCα) [31,32].

In addition to mediating PI3K-dependent signaling, AKT, PTEN and mTORC1/2 have also been shown to play a role in PI3K-independent signaling events (reviewed in [23,33,34,35,36]), and the PI3K-AKT-mTOR cascade interacts with multiple cooperative signal transduction cascades via a series of partially understood interactions and feedback loops to promote tumor growth (including MAPK, AR and WNT signaling, Figure 1). Hence, establishing the scope of this complex signaling program is fundamental for the identification of new and effective biomarkers and therapeutic approaches that will benefit patients with prostate cancer.

## 2. Genetic Aberrations in the PI3K-AKT-mTOR Pathway in Prostate Cancer Are Diverse

Augmented phosphorylation/activation of key PI3K-AKT-mTOR pathway components (e.g., p-AKT and p-mTOR) has been shown to correlate with prostate cancer progression in the clinic [37,38,39,40,41]. Furthermore, genomic and transcriptomic profiling has revealed that genetic alterations and deregulated gene expression of PI3K pathway components are common in patients with prostate cancer, occurring in as many as 42% of primary and 100% of metastatic prostate cancer samples [42,43,44,45,46]. Deregulation of the PI3K-AKT-mTOR pathway reflects a variety of genetic alterations, primarily PTEN loss-of-function [42,43,44,45,46]. To improve our understanding of the frequency and diversity of PI3K-AKT-mTOR pathway genetic aberrations in prostate cancer, we used the cBioPortal platform to survey three publicly available prostate cancer genomic datasets with primary and/or metastatic patient samples for a panel of 68 genes that encode key PI3K cascade components/effectors [47,48]. OncoPrints displaying the percentage frequency of each type of genetic aberration assessed within each dataset (i.e., gene mutation, amplification and deep deletion) highlight that PI3K-AKT-mTOR pathway genetic alterations are commonplace in primary and metastatic prostate cancer, and illustrate that the wide range of genetic events observed have a tendency to co-occur (Figures S1–S3 and Tables S1–S3, summarized in Table 1).

Common genetic alterations within the three prostate cancer datasets analyzed were observed in *PTEN*, *DEPTOR*, *SGK3*, *FOXO1/3*, *MAP3K7*, *RRAGD*, *SESN1*, *PIK3CA*, *PIK3C2B,* and *PDPK1* (Table 1). In addition, a vast range of less frequent aberrations were also detected, including genes encoding AMPK subunits (e.g., amplification of *PRKAB1* and *PRKAB2*) and AMPK regulators (e.g., *CAMKK2* and *LKB1* deletion) (Figures S1–S3, Tables S1–S3), as described below.

### 2.1. PI3K Gain of Function

#### 2.1.1. Class IA PI3Ks

Gain-of-function mutations in *PIK3CA* (encoding p110α) that activate the PI3K cascade are highly prevalent in a number of malignancies, including up to 40% of breast cancer patients [49] and as many as 53% of endometrial cancer patients [50]. In the prostate cancer datasets analyzed, *PIK3CA* mutation and high-level gene amplification occur in up to 4% and 9% of cases respectively (Tables S1–S3), although high-level amplification has been observed previously in as many as 29% of cases [13]. Our recent work identified that *PIK3CA* genetic alterations significantly correlate with poor prostate cancer prognosis, and that *Pik3ca* oncogenic mutation at a clinically relevant hotspot (H1047R) in mouse prostate epithelium can cause locally invasive prostate adenocarcinoma, demonstrating *Pik3ca* activation is a genetic driver of prostate cancer in vivo [13]. Although less common, *PIK3CB* mutation and amplification have also been detected in clinical prostate tumor specimens (0.6–1.8% and 1.8–3.1% respectively, Tables S1–S3), and activation of p110β (encoded by *PIK3CB*) predisposes prostate intra-epithelial neoplasia in mice [51]. Previous work has shown that p110α isoform-specific PI3K inhibitors can suppress *Pik3ca* mutant prostate cancer, whereas a p110β/δ inhibitor, or combined p110α/β blockade improves therapeutic outcome in *Pten*-deficient (p110β-dependent) prostate cancers [13,52,53]. Consequently, these findings have identified that selected p110 isoform-specific inhibitors may prove to hold efficacy against *PIK3CA* mutant and *PTEN*-deleted prostate cancer in the clinic.

Unlike the ubiquitous p110α and p110β PI3K catalytic isoforms, p110δ is predominantly expressed in cells of hematopoietic lineage and sensory neurons [54,55,56], and p110δ isoform-specific inhibitors are currently being explored in the clinic for B-cell malignancies and some autoimmune diseases [57]. However, several epithelial malignancies have also been shown to express p110δ [58], and 3% of patients with head and neck, germ cell, or colorectal cancer are reported to carry a *PIK3CD* mutation [59]. In patients with prostate cancer, *PIK3CD* mutation and amplification are infrequent events (≤1.1%, Tables S1–S3). However, a *PIK3CD* splice variant missing exon 20 (*PIK3CD-S*) has been identified in African American prostate cancer patients that can promote proliferation and AKT-mTOR signaling [60], and several CRPC cell lines have been shown to express p110δ at high levels, comparable to that detected in leukocytes [58]. In this study, inactivation of p110δ in p110δ-high CRPC cells suppressed PI3K-AKT signaling and inhibited cell proliferation, suggesting p110δ inhibitors may prove to hold therapeutic efficacy against p110δ-high prostate cancer [58].

The p85 regulatory subunit of PI3K is often present in a monomeric free form, and at a higher ratio relative to the p110 catalytic subunit, which suppresses p110 activity in the absence of stimuli [20,61,62]. Both free p85 monomers and p85–p110 heterodimers have been shown to bind to insulin receptor substrate (IRS), a cytoplasmic adaptor protein for the RTK insulin growth factor 1 (IGF-1) receptor, in addition to directly binding with activated RTKs [20,61]. Nonetheless PI3K-AKT-mTOR signaling activation is considered to require p85–p110 heterodimerization [20,61]. Interestingly, constitutive heterozygous deletion of *PIK3R1* (that encodes p85α and splice variants p55α/p50α) has been shown to lower blood glucose, enhance insulin sensitivity, potentiate insulin–stimulated glucose transport in skeletal muscle and adipocytes, and can stimulate insulin-dependent AKT phosphorylation in mouse liver [63,64]. Furthermore, liver-specific deletion of *Pik3r1* in mice is reported to not only enhance insulin and growth factor signaling, but causes development of aggressive hepatocellular carcinomas with pulmonary metastases associated with AKT activation and decreased PTEN expression [65]. *PIK3R1* shRNA-mediated knockdown in human breast cancer cell lines can also augment AKT signaling and anchorage-independent growth, illustrating a tumor suppressive role for p85α in breast cancer [66]. While p85α is generally viewed as a tumor suppressor, evidence in the literature also points toward an oncogenic role, similarly to p85β and p55γ [67,68,69,70,71].

In prostate cancer, *PIK3R1* is rarely mutated (0.4–1.6% of cases) yet deep deletions occur in 1–6% of patients (Tables S1–S3), which could potentially promote PI3K-AKT-mTOR signaling. Genetic alterations in *PIK3R2* (percentage incidence: mutation < 1.1%, amplification < 2.9%, deletion < 0.23%, Tables S1–S3) and *PIK3R3* (percentage incidence: mutation < 0.7%, amplification < 0.5%, deletion < 1%, Tables S1–S3) are also infrequent, yet the functional significance of these events remains unclear. Interestingly, down-regulation of *PIK3R1* in prostate cancer has been linked to reciprocal negative feedback between the AR and PI3K signaling cascades [72], and *PIK3R3* upregulation has been linked to prostate hyperplasia [73]. Furthermore, *PIK3R2* upregulation in prostate cancer specimens has recently been shown to inversely correlate with miR-126 expression [74]. Song and colleagues identified *PIK3R2* as a direct target of miR-126 in prostate cancer cell lines, and reported that enforced miR-126 expression in prostate cancer cell lines reduces *PIK3R2* mRNA expression and suppresses cell proliferation, migration, and invasion [74].

#### 2.1.2. Class IB PI3Ks

The smaller Class IB PI3K family is comprised of the catalytic subunit p110γ and two regulatory subunits, p101 and p87 (also known as p84), which are encoded by *PIK3CG*, *PIK3R5,* and *PIK3R6* respectively. Like Class IA, Class IB PI3Ks generate PIP3 from PIP2 to stimulate downstream effectors [19]. Class IB PI3Ks transmit Gβγ-GPCR and RAS signals to coordinate immune, inflammatory and allergic responses, predominantly within hematopoietic cells [18,19,20,22]. However, Brazzatti and colleagues have shown that knockdown of p110γ or p101 in 4t1.2 and MDA-MB-231 triple-negative breast cancer cell lines reduces migration in vitro and metastatic potential in xenograft mouse models, whereas p87/p84 knockdown had the opposite effect [75]. *PIK3CG* mutation and amplification are frequent in multiple malignancies, including 9–11% of melanomas and uterine, stomach and squamous cell lung cancers, while genetic alterations in *PIK3R5* and *PIK3R6* are prevalent in uterine cancer and melanoma, occurring in 4–8% of cases [50,76,77,78,79]. In prostate cancer, Class IB PI3K genetic aberrations are less common, and include *PIK3CG* mutation and amplification (1.4–1.8% and 0.6–3.6% incidence respectively) as well as *PIK3R5* and *PIK3R6* deep deletions (0–3.3% incidence) that are indicative of a homozygous deletion (Tables S1–S3).

#### 2.1.3. Class II PI3Ks

In comparison to Class I PI3Ks, the Class II family of PI3Ks (PI3KC2α, β and γ, encoded by *PIK3C2A*, *PIK3C2B,* and *PIK3C2G* respectively) is less well-characterized. Class II PI3Ks are generally considered to catalyze the production of lipid secondary messengers phosphatidylinositol 3-phosphate (PtdIns3*P* or PI(3)P) and phosphatidylinositol 3,4-bisphosphate (PI(3,4)P2) to mediate cell migration, channel regulation, endocytosis, and exocytosis [18,80]. The frequency of *PIK3C2A*, *PIK3C2B* and *PIK3C2G* mutation is generally low (0.2–1.4% incidence, Tables S1–S3), however *PIK3C2B* amplification has been observed in as many as 10% of cases (Table S3). Although the role of *PIK3C2B* amplification in prostate cancer is not clear, a recent study identified that PI3KC2β is highly expressed in PTEN-negative PC3 and LNCaP prostate cell lines compared to PTEN-positive DU145 prostate cancer cells (*PTEN*^+/−^), and PNT2 immortalized “normal” prostate epithelial cells (*PTEN^+/+^*) [81]. This study also reported that PI3KC2β regulates MAPK signaling to mediate prostate cancer cell invasion, thus the PI3KC2β-MEK-ERK signaling axis may present a novel therapeutic target for invasive prostate cancer [81].

#### 2.1.4. Class III PI3Ks

The Class III PI3K subfamily is comprised of the catalytic subunit vacuolar protein sorting 34 (VPS34) encoded by *PIK3C3*, and the regulatory subunit vacuolar protein sorting 15 (VPS15, or p150) encoded by *PIK3R4*. VPS34 catalyzes the phosphorylation of phosphatidylinositol (PI) to produce PI(3)P, which plays a central role in the regulation of intracellular trafficking [82]. To regulate the fusion and maturation of endosomes, VPS34 binds to VPS15 and Beclin-1 to form either VPS34 Complex I or VPS34 Complex II that differ by binding to Autophagy Related 14 (ATG14) or UV radiation resistance associated protein (UVRAG) respectively [83]. The Class III PI3K family has also been shown to mediate autophagy, endosome–lysosome maturation, membrane trafficking, and AMPK-dependent insulin sensitivity [82,84,85,86,87,88,89].

*PIK3C3* mutations are most frequently observed in uterine and gastric cancer patients (7% and 3.5% respectively), and *PIK3R4* gene mutation or amplification occur in up to 10% of squamous cell lung cancer and uterine cancer patients [50,76,77]. Although *PIK3C3*/*PIK3R4* mutation and *PIK3C3* gene amplification are infrequent events in prostate cancer (<1% of cases), *PIK3R4* high-level gene amplification is observed in up to 6.5% of cases and could potentially facilitate prostate cancer growth (Tables S1–S3).

Taken together, these data highlight the emerging diversity of genetic alterations within the PI3K family in prostate cancer, and emphasize the need for future work to gain further insight into the functional importance of these different genetic alterations during prostate cancer formation, progression, and recurrence. This is particularly important, as determining their non-redundant roles may present novel therapeutic targets and could aid patient stratification for future clinical trials.

### 2.2. Loss of Function of Phosphoinositide Phosphatases

Phosphoinositide phosphatases are a family of enzymes that dephosphorylate phosphoinositides to diminish phosphoinositide signals and regulate cellular functions [90]. The PI3K-AKT-mTOR pathway is regulated by multiple phosphoinositide phosphatases, including the tumor suppressor PTEN that dephosphorylates PIP3 into PIP2 to reduce PI3K-AKT-mTOR pathway activity (Figure 1). Genetic alterations in phosphoinositide phosphatases are strongly associated with human malignancies, and *PTEN* is one of the most frequently deleted genes in prostate cancer [91,92,93,94,95]. Here we review the frequency of genetic alterations in prostate cancer for genes encoding key phosphoinisitide phosphatases known to regulate the PI3K-AKT-mTOR cascade.

#### 2.2.1. Loss or Inactivation of PTEN

PTEN is a lipid/protein phosphatase that has been shown to negatively regulate the PI3K-AKT-mTOR pathway by dephosphorylating phosphatidylinositol (3,4,5)-trisphosphates (PIP3) back to phosphatidylinositol 4,5-bisphosphates (PIP2) (Figure 1) [23,96,97]. *PTEN* genetic alterations, primarily homozygous deletion, are common in advanced prostate cancer and significantly correlate with poor outcome and elevated PI3K-AKT-mTOR signaling [13,14,37,39,98,99]. The functional consequence of *PTEN* loss has been studied in vivo using a number of genetically engineered mice, which have demonstrated *PTEN* loss is a genetic driver of invasive prostate cancer [24,100,101,102,103,104]. Homozygous *Pten* deletion within the murine prostate epithelium leads to aggressive, locally invasive prostate carcinoma that has an inherent ability to acquire castration-resistant disease [13,24,100,101,102,105]. However, metastatic disease is rare in these models, possibly owing to the primary tumor reaching ethical limits before disseminated cells can colonize distant sites, differences in genetic background, and/or *PTEN* loss-induced p21/p53-dependent senescence [102,103,104,106,107].

In primary prostate adenocarcinoma, *PTEN* mutation and deep deletion occur in 2% and 18% of cases respectively (Table S1), and the frequency appears to increase in metastatic disease (6% and 26% respectively, Table S3). Although the majority of *PTEN* mutations identified in prostate cancer are truncating mutations, missense mutations are also observed, which could differentially impact PTEN lipid and/or protein phosphatase function [108]. Thus, determining how each PTEN genetic alteration impacts PTEN function may inform clinical trial design.

*PTEN* heterozygous deletion and epigenetic silencing can also deplete PTEN expression/function [92,98,106,109]. Importantly, mono-allelic deletion of *PTEN* has been reported in up to 68% of prostate cancer surgical specimens and PTEN immunohistochemistry (IHC) and/or fluorescent *in situ* hybridization analysis has revealed PTEN loss may occur in as many as 60% of advanced/CRPC cases [92]. A subset of patients with prostate cancer have also been found to harbor intratumoral heterogeneous *PTEN* loss [92], which could have significant implications for therapeutic strategies.

#### 2.2.2. Deregulation of Phosphoinositide Phosphatase Enzymes (other than PTEN)

In addition to PTEN, several other phosphatidylinositol phosphate phosphatase enzymes are also deregulated in human cancers that have the potential to facilitate malignant growth [90,91,110]. These phosphatases include; (a) proline-rich inositol polyphosphate 5-phosphatase (PIPP) encoded by polyphosphate-5-phosphatase J (*INPP5J*), (b) Src homology 2 (SH2) domain-containing inositol 5′-phosphatase 1 (SHIP1) encoded by inositol polyphosphate-5-phosphatase D (*INPP5D*), (c) Src homology 2 (SH2) domain-containing inositol 5′-phosphatase 2 (SHIP2) encoded by inositol polyphosphate phosphatase like 1 (*INPPL1*), and (d) inositol polyphosphate 4-phosphatase type II (INPP4B) encoded by *INPP4B*. While PTEN converts PIP3 to PIP2, PIPP and SHIP1/2 dephosphorylate PIP3 to phosphatidylinositol (3,4)-bisphosphate PI(3,4)P2, which is further hydrolyzed by INPP4B to form PI(3)P [90,111]. *INPP5D* deep deletion is observed in as many as 3.8% of patients with prostate cancer whereas *INPPL1* and *INPP4B* are amplified in up to 2.9% of cases (Tables S1–S3). *INPP5D*/*INPPL1*/*INPP5J*/*INPP4B* mutation, *INPP5J* amplification and *INPPL1*/*INPP4B/INPP5J* deep deletion events are rare (≤1.2%, Tables S1–S3). Relative to PTEN, the frequency of genetic alterations in these phosphoinositide phosphatases is much lower, however they are gaining increasing attention in the literature [111]. Interestingly, *PIPP* deletion is reported to increase tumor growth in the mouse mammary tumor virus-polyoma middle tumor-antigen (MMTV-*PyMT*) breast cancer model, and is accompanied with elevated proliferation, plasma membrane PIP3 levels, and AKT activation [110]. However, *PIPP* deletion also significantly reduced the incidence of lung metastasis in this setting, suggesting PIPP mediates a critical metastatic process [110,112]. Furthermore, INPP4B can compensate for PTEN loss by acting as a “back-up” phosphatase, and is regarded as a tumor suppressor in several epithelial tissues including the prostate, breast, ovary, and thyroid [112,113,114,115,116]. Notably, *Inpp4B* loss and *Pten* heterozygous deletion can cooperate in mice to facilitate metastatic thyroid cancer by increasing PIP3 levels and AKT signaling relative to single mutants [115], and enforced INPP4B overexpression in PC3 (*PTEN*^−/−^) and DU145 (*PTEN^+/−^*) prostate cancer cells can suppress prostate cancer cell migration and invasion, both in vitro and in vivo [117]. Immunostaining to detect INPP4B in prostate carcinoma clinical samples has also identified INPP4B loss as an independent prognostic marker, correlating with reduced biochemical (PSA) relapse-free survival [118]. In contrast, SHIP2 is reported to play an oncogenic role. Unlike PTEN that catalyzes PIP3 into PIP2, SHIP2 converts PIP3 into PI(3,4)P2 to further potentiate AKT activity [119,120]. Moreover, increased SHIP2 expression directly correlates with poor survival in patients with colorectal cancer [120]. Consequently, genetic aberrations in phosphoinositide phosphatase enzymes could prove to differentially influence therapeutic responses to PI3K pathway-directed therapies.

### 2.3. AKT Gain of Function

AKT isoforms 1, 2, and 3 (encoded by *AKT1*, *AKT2,* and *AKT3* respectively) form a subfamily of serine/threonine protein kinases that possess both overlapping and distinct cellular functions to regulate a variety of cellular processes during normal tissue homeostasis and cell transformation [121,122]. PI3K activity elevates PIP3 levels to recruit AKT to the plasma membrane where is it activated (Figure 1). AKT is activated by multiple kinases, including PDK1 and mTORC2 that phosphorylate AKT at residues Thr308 and Ser473 respectively, triggering a wave of phosphorylation through multiple downstream targets that stimulate cell survival, proliferation, metabolism and differentiation to promote tumor growth [19,20,32,123,124]. AKT downstream targets include PRAS40 (a component of mTORC1), BAD, FOXOs, and MDM2 (reviewed in [31]). AKT signaling is negatively regulated by several protein phosphatases that dephosphorylate and inactivate AKT, including protein phosphatase 2 (PP2A), and PH domain and leucine-rich repeat protein phosphatase-1 and -2 (PHLPP1 and PHLPP2) [125,126]. Below, we outline the various genetic alterations within the *AKT* isoforms and their regulators that have been detected in prostate cancer, and discuss their potential to activate AKT signaling and promote prostate tumor growth.

#### 2.3.1. AKT Mutation and Amplification

*AKT* genetic aberrations that increase AKT activity have been detected in multiple malignancies and are especially common in breast cancer, where *AKT3* amplification and *AKT1* E17K oncogenic mutation have been reported in up to 24% and 1–8% of cases respectively [127,128,129]. *AKT1*, *AKT2,* and *AKT3* activating mutations are rare in prostate cancer (≤0.9%, predominantly in *AKT1* at E17K), whereas *AKT1*, *AKT2,* and *AKT3* high-level gene amplification that can increase AKT activity is more common, particularly in advanced disease (up to 4.5%, 2%, and 4.7% incidence respectively, Tables S1–S3). Moreover, AKT activation in prostate cancer has been shown to positively correlate with Gleason score and invasive progression [37,130], and over-expression of myristoylated AKT (which causes constitutive AKT activation) causes prostate neoplasia in mice [131]. In support of an oncogenic role in prostate cancer and therapeutic resistance, conditional activation of AKT in either the LNCaP human prostate cancer cells or a transgenic mouse results in increased cell proliferation and inhibits cell death to promote tumor growth and castration-resistance in vivo [132]. Chen and colleagues have also demonstrated a requirement for AKT in *PTEN*-deficient prostate cancer, as *Akt1* haplodeficiency was found to suppress high-grade prostate intraepithelial neoplasia development within *Pten* heterozygous mice [133]. AKT inhibitors are being widely explored in the clinic to treat prostate cancer and have shown promise in *PTEN*-deficient patients [16,134].

#### 2.3.2. Genetic Alteration of AKT Regulators

A number of genetic alterations in genes that encode AKT regulators have been linked to prostate cancer, including kinases (e.g., PDK1), binding proteins (e.g., FKBP5), and phosphatases (e.g., PHLPP1, PHLPP2, and PP2A) [42,43,44,45,46]. PDK1 (encoded by *PDPK1*) is recruited to the membrane by PIP3 to phosphorylate and activate multiple targets, including AKT at residue T308 (Figure 1). *PDPK1* amplification and PDK1 over-expression are observed in several human cancers, including breast cancer [135]. In prostate cancer, *PDPK1* mutations are rare (≤0.2%), yet *PDPK1* amplification occurs in up to 8.1% of patients (Tables S1–S3). Interestingly, PDK1 RNAi-mediated knockdown does not impair *Pten*-deleted prostate cancer growth in mice, possibly reflecting mTORC2-mediated activation of AKT, and/or compensatory augmentation of the MAPK cascade [136]. These findings suggest that PDK1 inhibitors are not likely to be efficacious against *PTEN*-deficient prostate cancer in the clinic as a single agent.

*FKBP5* (also known as *FKBP51*) is an AR target gene that plays a key role in mediating the cellular distribution of steroid hormone receptors and has been shown to negatively regulate AKT signaling by stabilizing PHLPP1/2 (Figure 1) [11,24,137]. During androgen/AR-directed therapy, FKBP5-PHLPP1/2-AKT signaling forms a negative feedback loop between the AR and PI3K-AKT-mTOR pathways to facilitate ADT resistance [11,24,137], discussed in Section 3.2. Mutation and deep deletion of *FKBP5* are fairly infrequent in prostate cancer (≤1.22%, Tables S1–S3), however FKBP5 down-regulation has been linked to CRPC and increased AKT signaling [11].

PHLPP1 and PHLLP2 (encoded by *PHLPP1* and *PHLPP2)* are protein phosphatases that dephosphorylate and inactivate AKT. *PHLPP1* and *PHLPP2* deep deletion occurs in up to 3.9% and 6.5% of patients with prostate cancer respectively (Tables S1–S3), which could potentially sustain AKT-signaling. Interestingly, Chen and colleagues reported a strong tendency for *PTEN*, *PHLPP1*, *PHLPP2*, and *TP53* co-deletion in metastatic prostate cancer and that low *PHLPP1* expression correlates with reduced patient survival and relapse after surgery [138]. Additionally, the tumor suppressive function of PHLPP1 has been demonstrated in vivo, as *Phlpp1* loss causes prostate neoplasia in mice and promotes invasive carcinoma progression in *Pten^+/−^* transgenic mice [138]. In contrast, *Phlpp2* loss impairs *Pten*/*p53*-deleted prostate tumor growth in mice [139], indicating PHLPP1 and PHLPP2 mediate differential AKT-independent functions. Indeed, PHLPP2 can dephosphorylate MYC at residue Thr58 to prevent MYC degradation and promote tumor progression [139]. Consequently, in PHLPP2-positive MYC-driven advanced prostate cancer, it has been suggested that PHLPP2 may present a valuable therapeutic target [139].

In addition, genetic alterations in *PPP2CA* (protein phosphatase 2 catalytic subunit alpha) that encodes the negative AKT regulator PP2A have also been observed in prostate cancer [42,43,44,45,46], and PP2A loss has been linked to prostate cancer progression and metastatic potential in the clinic [140]. *PPP2CA* mutation and deep deletion events occur in 0.4–1.4% of patients with prostate cancer (Tables S1–S3), further highlighting the diversity of genetic aberrations in AKT regulators that could promote oncogenic PI3K signaling.

### 2.4. SGK Deregulation

The serum/glucocorticoid-regulated kinase isoforms SGK1, SGK2, and SGK3 belong to a subgroup of the AGC (cAMP-dependent, cGMP-dependent, and protein kinase C) family of protein kinases that play a role in multiple cellular processes including cell growth, proliferation, metabolism, intracellular trafficking and survival [141,142,143]. SGK1 and 3 are considered to be ubiquitously expressed, while SGK2 expression is prominent in the liver, kidney, pancreas, and brain [144]. SGKs share structural similarities, upstream regulators, substrates and functions with the AKT isoforms (reviewed in [142]). For instance, all SGKs are phosphorylated and activated by PDK1, and SGK1 is a downstream target of mTORC2 [26,143,145,146,147,148] (Figure 1). SGKs are also activated by PI3K/PDK1-independent mechanisms, for example SGK1 is regulated by big mitogen-activated protein kinase-1 (BMK-1) and p38 mitogen-activated protein kinase in response to epidermal growth factor (EGF) and interleukin-6 (IL6) respectively [149,150]. Although the role of SGKs during prostate cancer is currently unclear, SGK1 over-expression has been shown to facilitate CRPC transition in a prostate cancer xenograft model, indicating that SGK1 can promote ADT-resistance [151]. Furthermore, the SGK1 inhibitor GSK650394 has been shown to induce autophagy and apoptosis in PC3, LNCaP, DU145, and CWR22RV1 prostate cancer cells in vitro [152]. Interestingly, SGK1 and SGK3 have also been linked to PI3K/AKT-targeted therapy resistance in breast cancer [145,148]. Gasser and colleagues have also shown that INPP4B over-expression leads to enhanced SGK3 activation in ZR-75-1 breast cancer cells, triggering a switch from AKT- to SGK-dependent signaling downstream of PDK1 [153].

Mutation of the SGK isoforms is a rare event in human cancers, however gene amplification is commonly detected [154]. In keeping with this, *SGK1*, *SGK2,* and *SGK3* are rarely mutated in prostate cancer (≤0.41%), whereas amplification occurs in up to 2.5%, 2.0%, and 20.3% of cases respectively (Tables S1–S3). Of note, the frequency of *SGK3* gene amplification is particularly high in the SUC2/PCF IDT metastatic prostate adenocarcinoma dataset (Table S3), underlining the need for future studies to establish how SGKs contribute to prostate cancer and metastatic progression.

### 2.5. Loss of FOXO Transcription Factors

The mammalian forkhead box O (FOXO) family consists of four transcription factors (FOXO1, 3, 4, and 6) that are highly similar in structure and function [155]. In response to insulin and growth factors, FOXOs modulate the transcription of several target genes to mediate key cellular processes including proliferation, apoptosis, autophagy, inflammation, metabolism and stress resistance, and they form an important regulatory circuit within the AKT and mTOR signaling cascades [156,157,158] (Figure 1). FOXOs are regulated by several kinases, including AKT and SGK isoforms, which phosphorylate and inactivate FOXO-mediated gene transcription by inhibiting FOXO DNA binding and triggering FOXO nuclear-to-cytoplasm translocation [157,158,159]. FOXOs are generally regarded as tumor suppressors, and are reported to inhibit mTORC1 via sestrins, however a number of oncogenic functions are emerging in the literature [156,157,158]. For instance, FOXO-mediated transcription of the mTORC2 component *RICTOR* in response to physiological stress is reported to promote mTORC2 signaling [156]. FOXOs also provide a reciprocal negative feedback loop between PI3K-AKT-mTOR pathway and AR signaling [12] (discussed in Section 3.2).

In prostate cancer, FOXO mutations are rare (<0.5% incidence), however *FOXO1* and *FOXO3* deep deletion is a frequent event, occurring in up to 15.2% and 13.4% of patients respectively (Tables S1–S3). *FOXO3* lies within the 6q21 locus that is frequently lost in prostate cancer [160], and reduced FOXO3 (also FOXO3a) activity via peptide driven inhibition is reported to accelerate prostate cancer progression in the transgenic adenocarcinoma mouse prostate (TRAMP) neuroendocrine prostate cancer model [161]. FOXO1 has also been shown to bind and inhibit the transcriptional activity of E26 transformation-specific (ETS) transcription factor ERG, which is over-expressed in 50% of prostate cancers owing to TMPRSS2-ERG (transmembrane protease, serine 2: ERG fusion) gene rearrangements [162]. Furthermore, *Foxo1* bi-allelic deletion and ERG overexpression can cooperate to cause prostate neoplasia in mice [162]. Together, these findings suggest FOXO1/3 act as tumor suppressors during prostate cancer.

*FOXO4* gene amplification occurs in up to 8.8% of patients with metastatic prostate cancer (Table S3), however the functional importance of this genetic alteration remains to be clarified. Although *FOXO4* down-regulation is reported to correlate with reduced prostate cancer metastasis-free survival, conversely *FOXO4* knockdown in LNCaP cells can increase metastatic potential [163]. Thus, future work addressing the role of *FOXO4* during prostate cancer progression is warranted.

### 2.6. TSC1-TSC2-TBC1D7 Complex and RHEB Deregulation

To regulate mTORC1 signaling, TSC1, TSC2, and TBC1D7 form a complex to suppress RHEB GTPase, an upstream activator mTORC1 [164] (Figure 1). Activated AKT directly phosphorylates TSC2 at multiple residues to inhibit the TSC1:TSC2 complex, activate RHEB GTPase, and subsequently stimulate mTORC1 signaling [165,166]. TSC2 is also regulated by MAPK, WNT, and energy signals through coordinated phosphorylation by ERK, GSK3, and AMPK respectively, thus limiting mTORC1 activation and cell growth in response to poor growth conditions, and illustrating TSC2 as a central node for PI3K-AKT-mTOR crosstalk with multiple signaling cascades [164,166,167,168].

*TSC1* and *TSC2* are frequently mutated/deleted in a variety of solid tumors, including lung (22%) and liver (16%) cancers, leading to deregulated PI3K-AKT-mTOR signaling [169,170]. In prostate cancer, the frequency of *TSC1* and *TBC1D7* mutation or deep deletion is low (≤0.8% incidence, Tables S1–S3), whereas *TSC2* mutation and deep deletion are more frequent (1–1.8% and up to 4.2% of cases respectively, Tables S1–S3). Interestingly an inactivating splice variant of *TSC2* unique to African American patients with prostate cancer has also recently been linked to aggressive prostate cancer and therapeutic resistance [60]. In mice, *Tsc1* conditional deletion in murine prostate epithelium is reported to cause prostate neoplasia associated with elevated mTORC1 signaling [171], and combined *Tsc2* and *Pten* heterozygosity has been shown to promote invasive prostate carcinoma relative to single mutants [172]. In lung cancer, TSC1 and TBC1D7 have been shown to function as oncoproteins [173], possibly reflecting mTORC1-independent functions such as TSC1-mediated activation of TGFβ-SMAD2/3 signaling [174]. Remarkably, up to 3%, 4%, and 7% of patients with prostate cancer also display *TBC1D7*, *TSC1,* and *TSC2* high-level amplification respectively (Tables S1–S3), yet the functional consequence is currently unclear.

RHEB GTPase has also been shown to act as a proto-oncogene in prostate cancer and up to 4% of patients with prostate cancer carry *RHEB* gene amplification, however *RHEB* oncogenic mutations are rare (≤0.1% incidence, Tables S1–S3). RHEB GTPase is over expressed in several prostate cancer cell lines and transgenic mice over-expressing *Rheb* specifically within the prostate epithelium develop low-grade prostatic intraepithelial neoplasia lesions by 10 months of age, accompanied with increased mTORC1 activity [175]. *Rheb* over-expression can also cooperate with *Pten* haploinsufficiency to promote prostate tumorigenesis [175], indicating *RHEB* amplification is likely to be a genetic driver of prostate tumorigenesis in the clinic.

### 2.7. Amplification of mTORC1 and mTORC2 Complex Components

The mTORC1 and mTORC2 protein complexes are functionally and structurally distinct, originally distinguished by their sensitivity to the mTOR inhibitor rapamycin [176,177,178]. Both mTORC1 and mTORC2 complexes contain mTOR, MLST8 (also known as G-protein beta-subunit like, GβL), TEL2, TTI1, and the negative regulator DEPTOR [179,180]. RAPTOR and PRAS40 (encoded by *AKT1S1*) are additional members of mTORC1 complex, whereas RICTOR, mSIN1, and PROTOR1/2 form the mTORC2 complex [180] (Figure 1). mTORC1 and mTORC2 are downstream effectors and regulators of PI3K/AKT signaling that mediate key cellular processes in response to growth factors and hormones [179,181,182,183]. mTORC1 is sensitive to rapamycin treatment and functions to regulate cell growth, autophagy, protein translation machinery, and cell-cycle progression by phosphorylating substrates such as ULK1, S6K and 4EBP1 [179,183,184,185]. The mTORC2 complex plays a critical role in PI3K/AKT signaling by increasing the activity of AKT, SGK1 and PKCα to regulate cell survival, metabolism and cytoskeletal dynamics [184] (Figure 1). mTORC2 is generally insensitive to rapamycin [179], however chronic exposure to the drug has been shown to impair mTORC2 assembly [185]. Crucially, mTORC1 and mTORC2 can also regulate each other via multiple mechanisms, including AKT regulation of PRAS40 to block suppression of mTORC1 activity and S6K regulation of mSIN1 to modulate mTORC2 activity [186].

In general, the frequency of genetic alterations in mTORC1 and mTORC2 components is low in prostate cancer. Genomic profiling data have shown that *mTOR* mutation occurs in 0.6–1.6% of cases, and the frequency of mutation or deep deletion in the other components of mTORC1/2 is ≤1% (Tables S1–S3). However, *DEPTOR* gene amplification is comparatively frequent, occurring in 5.1–21.4% of cases, with the highest incidence observed in the SUC2/PCF-IDT metastatic prostate adenocarcinoma dataset (Tables S1–S3). In addition, *DEPTOR* amplification directly correlates with worse disease/progression-free survival in the TCGA Firehose Legacy prostate adenocarcinoma dataset (Figure S4), indicating *DEPTOR* amplification may provide a valuable predictive biomarker in the clinic. DEPTOR is an endogenous suppressor of mTOR kinase activity, yet *DEPTOR* upregulation can reduce S6K1 activation, thus relieving feedback inhibition from mTORC1 to PI3K and mTORC2 signaling that results in increased AKT activation [187]. Nevertheless, *DEPTOR* knockdown in colorectal cancer cells reduced cell proliferation and induced differentiation [188], raising the possibility that *DEPTOR* can promote tumorigenesis in other epithelial cancers. DEPTOR has also been shown to exert mTORC1/2-independent functions in the nucleus as a transcriptional regulator in multiple myeloma cells [189] and is a transcriptional target of WNT/β-catenin/MYC signaling in colorectal cancer cells [188], adding further complexity to PI3K-AKT-mTOR and WNT pathway crosstalk.

In addition to *DEPTOR*, a number of other genes encoding mTOR components were also distinctly amplified in the SUC2/PCF-IDT metastatic prostate cancer dataset (*AKT1S1*, 2.7%; *MLST8*, 7.7%; *MAPKAP1*, 4.5%; *RPTOR*, 7%; *RICTOR*, 5%; *TELO2*, 6.5%; *TTI1*, 2.5%, Table S3), which could potentially facilitate tumor progression. However, none of these genetic alterations correlate with disease/progression-free survival (determined by cBioPortal analysis of the TCGA Firehose Legacy prostate adenocarcinoma dataset, *n* = 492, data not shown) [47,48]. Significantly, bi-allelic deletion of *Rictor* in mouse prostate epithelium has revealed RICTOR is not required for normal tissue homeostasis, yet RICTOR loss can suppress *Pten*-deleted prostate tumorigenesis in mice [190]. These findings indicate that mTORC2 signaling can contribute to *PTEN*-deleted prostate cancer growth, and that mTORC2 inhibition may be efficacious in the clinic against prostate cancers with *PTEN* loss [190].

Intracellular amino acids can also activate mTORC1 signaling by stimulating vacuolar H^+^-ATPase (v-ATPase) to activate Ragulator, a guanine exchange factor that converts RAGA/B·GDP to RAGA/B·GTP, enabling formation of the active RAG complex where RAGA·GTP or RAGB·GTP form heterodimers with either RAGC·GDP or RAGD·GDP [36,191,192,193,194,195]. Similarly to RAGA/RAGB, RAGC/RAGD are functionally redundant and are 80–90% homologous [195]. When amino acids are sufficient, mTORC1 is recruited to the lysosome where it binds to the active RAG complex via RAPTOR, followed by its localization to RHEB that leads to mTORC1 activation [36,191,195] (Figure 1). Recent evidence also suggests that amino acids such as glutamine can activate mTORC1 in a RAG-complex independent manner, for example via the GTPase adenosine ribosylation factor 1 (ARF1) [196], highlighting the complex nature of mTORC1 regulation.

In prostate cancer, genetic alterations in *RRAGA* and *RRAGC* genes that encode RAGA and RAGC respectively are uncommon, however *RRAGB* (encoding RAGB) is amplified in up to 7.7% of cases and *RRAGD* (encoding RAGD) deep deletion occurs in 6.5–14.4% of cases (Tables S1–S3). Interestingly, *RRAGD* deep deletion in prostate adenocarcinoma strongly correlates with *FOXO3* deletion (one-sided Fisher’s Exact test, *p*-value < 0.001; data sourced from the cBioPortal platform, TCGA Firehose Legacy prostate adenocarcinoma dataset, *n* = 492), however the functional consequence of *RRAGD*/*FOXO3* co-deletion and *RAGB* amplification during prostate cancer growth and therapeutic resistance is currently unknown and merits further investigation.

### 2.8. Aberrant AMPK Signaling

The metabolic sensor AMPK functions to maintain an adenosine triphosphate (ATP) equilibrium, influencing cell growth, lipid and glucose metabolism, autophagy and cell polarity [197]. AMPK is composed of a catalytic subunit (α1/α2, encoded by *PRKAA1*/*PRKAA2*), a β structural subunit (β1/β2, encoded by *PRKAB1*/*PRKAB2*) and a regulatory γ subunit (γ1/γ2/γ3, encoded by *PRKAG1*/*PRKAG2*/*PRKAG3*) [198]. AMPK activation plays a tumor suppressive role by inhibiting mTORC1 through the phosphorylation of TSC2 and RAPTOR in response to energy stress [199] (Figure 1), and by negatively regulating lipogenesis [200,201,202]. AMPK can also play an oncogenic role during stress (including hypoxia, oxidative stress, and glucose deprivation) to activate AKT, yet the molecular mechanisms involved remain to be fully elucidated [200]. Mutation and deep deletion of the AMPK subunits are uncommon in human malignancies [203], including prostate cancer (<1.2% incidence, Tables S1–S3). Instead, gene amplification of the AMPK subunits is more common [43,45,204]. In prostate cancer, high-level amplification of *PRKAB1*, *PRKAB2*, *PRKAG2*, and *PRKAG3* occurs in up to 6.3%, 6.8%, 4.1%, and 2% of cases respectively (Tables S1–S3). Whether AMPK amplification equates to increased activity remains to be determined, however AMPK phosphorylation/activation is reported to positively correlate with Gleason score and disease progression [205,206].

Interestingly, androgen-mediated activation of AMPK has been shown to increase the growth of prostate cancer cells, associated with elevated intracellular ATP levels and peroxisome proliferator-activated receptor gamma coactivator 1-alpha (PGC-1α)-mediated mitochondrial biogenesis [206]. Thus, AR-mediated AMPK activation could potentially function to avoid energy crisis and promote tumor growth. Upstream activators of AMPK include Ca^2+^/calmodulin-dependent protein kinase kinase β (CAMKKβ), liver kinase B1 (LKB1), sestrins, and potentially mitogen-activated protein kinase kinase kinase 7 (MAP3K7) [207,208,209]. Below we explore several potential mechanisms underpinning deregulation of the AMPK-AKT/mTOR signaling axis in prostate cancer.

#### 2.8.1. CAMKKβ Amplification

CAMKKβ is encoded by *CAMKK2* and phosphorylates AMPK in response to Ca^2+^ signaling. In prostate cancer, *CAMKK2* is amplified in up to 6.3% of patients (Tables S1–S3), however it is currently unknown if *CAMKK2* amplification promotes AMPK activity in the clinic. In a *Pten*-deleted prostate cancer mouse model, *Camkk2* deletion or CAMKKβ pharmacological inhibition has been shown to suppress prostate tumorigenesis and reduce *de novo* lipogenesis, whereas *Prkab1* (AMPK-β1) and *Pten* co-deletion accelerates tumor progression [210]. These findings indicate that CAMKKβ plays an oncogenic role in this setting and that CAMKKβ and AMPK-β1 play opposing roles in *Pten*-deficient prostate cancer, possibly reflecting their differential regulation of lipogenesis [210]. CAMKKβ has also been shown to activate AMPK in response to androgen signaling, and AMPK can subsequently inhibit AR function to form a negative feedback loop [210]. However, the impact on the PI3K-AKT-mTOR signaling cascade remains unclear. Interestingly, a recent report has shown that CAMKKβ can directly phosphorylate AKT at residue Thr308 in ovarian cancer cells [211], indicating CAMKKβ may regulate AKT/mTOR signaling both directly and indirectly via AMPK.

#### 2.8.2. LKB1 Loss

LKB1 (encoded by serine/threonine kinase 11, *STK11*) is a multifaceted enzyme that plays a tumor suppressive role by phosphorylating multiple substrates (e.g., AMPK and PTEN) to regulate crucial cellular processes including cell metabolism, polarity, differentiation, and proliferation [212,213] (Figure 1). While *STK11* deletion or inactivating mutations are frequent in lung cancer (occurring in up to 50% of patients) [208], *STK11* mutations are rare in prostate cancer (0.2% incidence, Tables S1–S3) and the frequency of *STK11* deep deletion is also comparatively low (0–3.4% incidence, Tables S1–S3). We have previously shown that LKB1 exerts a tumor suppressive function in the prostate, as *Lkb1* homozygous deletion in murine prostate epithelial cells causes prostate intra-epithelial neoplasia (PIN), associated with elevated PI3K/AKT signaling [214]. The relatively mild effects of LKB1 loss are greatly enhanced when combined with *Pten* heterozygosity in the mouse prostate, which causes lethal metastatic prostate cancer [215]. Interestingly, the expression of either wild-type LKB1, or a kinase-dead form of LKB1 (LKB1^K78I^) is sufficient to reduce tumor burden and impair metastatic potential of DU145 prostate cancer cells that lack LKB1, indicating LKB1 may also elicit a kinase-independent tumor suppressive function [215]. These in vivo findings indicate that deregulation of the LKB1-AMPK signaling axis is a potential mechanism whereby AKT/mTOR signaling is potentiated to facilitate prostate tumor formation and/or progression. Furthermore, a recent study has shown that LKB1 protein levels are reduced in immortalized prostate cancer cell lines relative to normal prostate epithelial cells, and siRNA-mediated *STK11* knockdown correlated with elevated hedgehog signaling and increased proliferation and invasion of prostate cancer cells in vitro, however PI3K-AKT-mTOR signaling was not assessed [216].

#### 2.8.3. Sestrin Deletion

Sestrins are a family of stress inducible antioxidant proteins comprising of SESN1, SESN2, and SESN3, which play a key role in regulating autophagy, mitophagy, metabolic homeostasis, inflammation, hypoxia and oxidative stress [217,218,219]. SESN1 and SESN2 are p53 target genes that are induced upon DNA damage and oxidative stress [217]. SESN1 and SESN2 can directly bind to both the TSC1:TSC2 complex and AMPK, which leads to AMPK activation/autophosphorylation in a p53-dependent manner and stimulates AMPK-mediated phosphorylation of TSC2 to negatively regulate mTORC1 signaling [217]. In addition, sestrins are reported to negatively regulate mTORC1 signaling via GATOR2/RAG, indicating that sestrins can also mediate PI3K-AKT-mTOR signaling in response to energy stress (e.g., nutrient starvation) [220,221].

Genetic alterations in the genes encoding sestrins have been linked to non-small cell lung carcinoma (NSCLC) and colorectal cancer, and recent evidence in the literature has indicated sestrins play a tumor suppressive role [221,222]. Although sestrin mutations are rare (≤0.8%), *SESN1* deep deletion is a frequent event in prostate cancer occurring in 4.7–13.4% of cases (Tables S1–S3), potentially leading to increased mTORC1 signaling through alleviation of SESN1-mediated negative regulation of mTORC1. Interestingly, similarly to *FOXO3*, *SESN1* is located within the 6q21 locus that is commonly lost in prostate cancer [160]. *SESN1* is also reported to be transcriptionally repressed by AR [223], whereas p53 and FOXOs are known to mediate *SESN1* transcription [156,217]. Thus, future work exploring the functional significance and predictive value of *SESN1* depletion in prostate cancer could identify new therapeutic avenues or biomarkers to aid patient care.

#### 2.8.4. MAP3K7 Deletion

MAP3K7 (also known as transforming growth factor (TGF) β-activated kinase 1, TAK1) is a serine/threonine protein kinase that mediates cell survival via NF-κB-dependent and NF-κB-independent signaling in response to TGFβ and cytokines [224]. Recent evidence in the literature has indicated that MAP3K7 may also mediate AMPK-AKT-mTOR signaling, as MAP3K7/TAK1 inactivation is associated with AMPK activation and reduced p-mTOR levels in skeletal muscle [209]. However, MAP3K7 is reported to mediate mTOR signaling independently of AMPK in hepatocellular carcinoma, possibly via p38 activation [225].

In prostate cancer, *MAP3K7* is a putative tumor suppressor gene and *MAP3K7* deletion has been shown to directly correlate with prostate cancer progression, lymph node metastasis, and biochemical recurrence [226,227]. *MAP3K7* deep deletion is a frequent event in prostate cancer, occurring in up to 14.8% of patients (Tables S1–S3). Furthermore, loss of *Map3k7* in mice has been shown to promote prostate tumorigenesis [227], suggesting MAP3K7 plays a tumor-suppressive function in the prostate. However, in an AML xenograft model MAP3K7 inhibition was found to attenuate leukemia development [228], indicating that MAP3K7 plays a dual role as a tumor suppressor and an oncogene depending on the malignancy.

## 3. The PI3K-AKT-mTOR Pathway Intersects with Multiple Oncogenic Signaling Cascades to Facilitate Prostate Cancer Growth

The PI3K-AKT-mTOR signaling cascade is one of the most frequently upregulated pathways in prostate cancer, which potentiates multiple downstream signaling events to mediate a plethora of cellular processes that promote tumor growth and therapeutic resistance to current treatment regimens. Targeting the PI3K-AKT-mTOR pathway using small molecules, such as pan-PI3K, PI3K-isoform specific, AKT, mTOR and dual PI3K/mTOR inhibitors has been challenging owing to their limited efficacy and poor tolerability (reviewed in [14,15,16,17,134,229,230]). Many clinical trials involving PI3K-AKT-mTOR-directed therapies have failed owing to incomplete inhibition of the pathway, reflecting the multiple modes of pathway redundancy and numerous positive/negative feedback loops that exist both within the PI3K-AKT-mTOR cascade and via crosstalk with other signaling pathways [15,231,232,233,234,235,236] (Figure 1). Here, we review PI3K-AKT-mTOR interactions with the RAS/MAPK, AR, and WNT signaling pathways, illustrating the need to improve our molecular understanding of the broader PI3K-AKT-mTOR signaling network. Delineating the complexity of the PI3K-AKT-mTOR pathway interactions with other signaling cascades during normal tissue homeostasis, tumorigenesis and therapeutic resistance is crucial for the discovery of new, efficacious personalized treatment approaches that overcome PI3K-AKT-mTOR inhibitor resistance.

### 3.1. PI3K-AKT-mTOR and RAS/MAPK Signaling Crosstalk

The RAS/MAPK cascade transduces extracellular growth signals via transmembrane receptors (e.g., RTKs and GPCRs) and a series of intracellular protein kinases to regulate gene expression in the nucleus, and to mediate a range of cellular functions including cell proliferation, migration, differentiation, senescence, and survival [25,237,238]. Growth factors bind to the extracellular surface of RTKs (e.g., epidermal growth factor receptor, EGFR, and fibroblast growth factor receptor, FGFR) leading to a conformational change that enables RTK dimerization and autophosphorylation of several tyrosine residues within the RTK cytoplasmic tail. This creates docking sites for adaptor proteins that stimulate downstream effector cascades, such as growth factor receptor-bound protein 2 (GRB2) that recruits Son of Sevenless (SOS) and the GTPase RAS to activate the MAPK cascade (RAF-MEK-ERK signaling) and drive transcription of RAS/MAPK target genes [237,238] (Figure 1).

The RAS/MAPK cascade is frequently deregulated in human cancers, including prostate cancer [238]. Activating genetic alterations (i.e., mutation/amplification) in *RAS (HRAS*, *NRAS,* or *KRAS)* and *BRAF* have been reported in primary and metastatic prostate cancer (1–8% incidence), and augmented MAPK signaling is reported to correlate with castration-resistance and metastatic progression [43,45,46,101,239]. The PI3K-AKT-mTOR and RAS/MAPK pathways are interconnected at multiple levels (Figure 1), predominantly owing to (a) shared upstream regulation mechanisms through RTKs/GPCRs and their associated adaptors, (b) the ability of respective cytosolic signaling components to interact and cross-regulate, and (c) the regulation of joint downstream targets (e.g., BAD and RPS6), reviewed in [25,240]. At the level of the receptor for example, the GRB2-SOS complex that is recruited to activated RTKs can bind to the scaffolding protein GAB1 (GRB2-associated binder-1), which interacts with RasGAP, SHP2, PI3K, and PIP3 to augment both RAS/MAPK and PI3K-AKT-mTOR signaling [25]. In addition, mTORC1 signaling can negatively regulate RTK signaling to reduce both PI3K-AKT-mTOR and RAS/MAPK activity, including mTORC1-S6K-mediated suppression of the insulin receptor substrate protein IRS1; a major IGF-1 receptor substrate and adaptor protein that can promote both PI3K and RAS activation by binding to p85 and GRB2 respectively [25,241]. S6K can also phosphorylate RICTOR to reduce mTORC2 signaling [25,242].

At the membrane, RAS-GTP can also bind to the RAS-binding domain (RBD) of p110α, p110δ, and p110γ to directly activate several Class I PI3K catalytic subunit isoforms [20,243]. Intracellular components of both cascades also interact to form multiple feedforward and feedback loops that enable PI3K-AKT-mTOR and RAS/MAPK pathway cross-regulation (Figure 1) [25,232,240]. For instance, RAS/MAPK activation has been shown to stimulate mTORC1 signaling through ERK, which can directly phosphorylate TSC2, RAPTOR and 90 kDa ribosomal S6 kinase (RSK) to inactivate/dissociate the TSC1:TSC2 complex and regulate the recruitment of mTORC1 substrates [244,245,246]. ERK-RSK signaling can also phosphorylate serum response factor (SRF), cAMP response element-binding protein (CREB) and RPS6, thus promoting cap-dependent translation independently of mTORC1-S6K signaling [247,248]. In addition, AKT is also reported to directly phosphorylate and negatively regulate RAF to suppress the MAPK cascade [249,250], and activated RAS has recently been shown to directly interact with mSIN1 to stimulate mTORC2 signaling in cancer cells (including prostate cancer cell lines) [251].

#### 3.1.1. RAS/MAPK-PI3K-AKT-mTOR Interactions Promote Resistance to PI3K-AKT-mTOR Pathway-Directed Therapies

**Figure 2:**
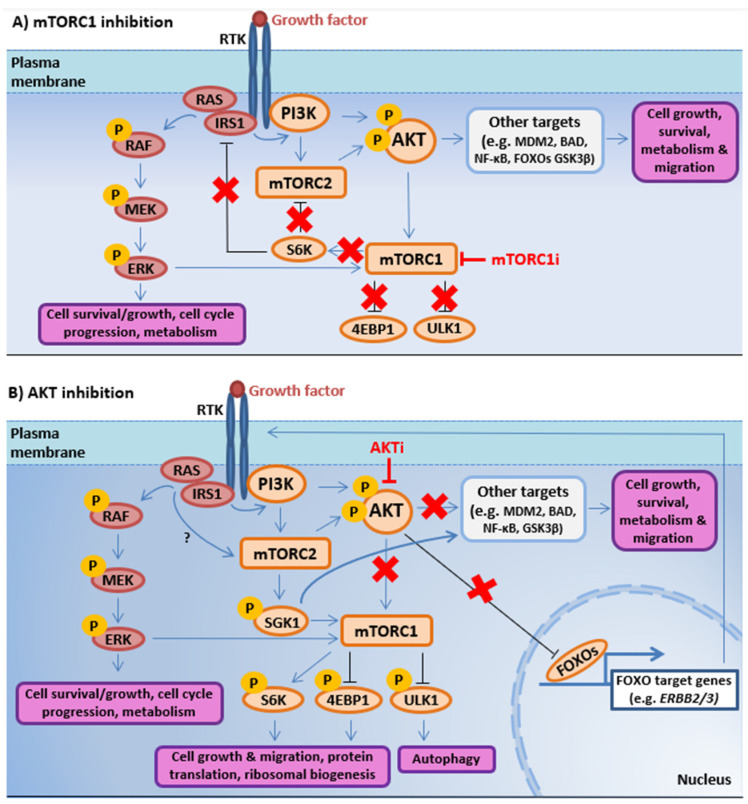
PI3K-AKT-mTOR and RAS/MAPK pathway crosstalk can contribute to mTORC1 and AKT inhibitor resistance. Model schematics illustrating reported mechanisms of therapeutic resistance to (**A**) mTORC1 inhibition and (**B**) AKT inhibition. mTORC1 and AKT blockade potentiates a series of feedback/feedforward loops between the PI3K-AKT-mTOR and RAS/MAPK signaling pathways, leading to augmented RAS/MAPK signaling and incomplete suppression of the PI3K-AKT-mTOR cascade that can promote drug-resistant tumor growth. AKTi, AKT inhibitor; mTORC1i, mTORC1 inhibitor.

Clinical trials exploring the efficacy of inhibitors targeting the PI3K-AKT-mTOR pathway in prostate cancer have been extensively reviewed previously [14,15,16,17]. Despite promising results in early preclinical studies [252,253], allosteric mTORC1 inhibitors (e.g., rapamycin and rapamycin analogs/rapalogs such as Everolimus and Temsirolimus) have been ineffective in patients with prostate cancer, owing to their inability to suppress AKT activity and a number of adverse side effects [17,254]. Evidence in the literature has revealed several mechanisms of resistance, including activation of the RAS/MAPK pathway [235,255,256,257]. Both normal and transformed prostate epithelial cells have been shown to augment RAS/MAPK signaling in response to mTORC1 inhibition [235,255], and administration of Everolimus (RAD001) has been shown to induce MAPK signaling in a *Pten*-deleted mouse model of prostate cancer [235,255]. Although the mechanisms underpinning resistance to mTORC1 inhibitors are not completely understood, several signaling events that involve PI3K/AKT/PI3K and RAS/MAPK crosstalk have been identified. For instance, mTORC1 inhibition is reported to promote AKT and RAS/MAPK signaling by blocking mTORC1-S6K-mediated negative regulation of IRS1 and mTORC2 signaling [258,259] (Figure 2A). Inhibition of mTORC1 has also been shown to prevent mTORC1 stabilization of growth factor receptor bound protein 10 (GRB10), an RTK adaptor protein that negatively regulates RTK signaling [260].

Resistance to AKT inhibitors (e.g., capivasertib and ipatasertib) has also been linked to elevated RAS/MAPK signaling and mTORC2 activity [14,261]. AKT inhibition can lead to the nuclear accumulation of active FOXO1, resulting in increased transcription of FOXO1-regulated genes, such as *ERBB2/3* that encode human epidermal growth factor 2/3 (HER2/3) RTKs [25,237,262,263,264] (Figure 2B). PI3K inhibition with either pan-PI3K inhibitors (GDC0941 and XL-147) or a dual PI3K/mTOR inhibitor (BEZ235) has also been found to increase HER2/3 expression in breast cancer, resulting in increased RAS/MAPK signaling [265,266]. Furthermore, FOXO-dependent transcription is associated with p110α and PDK1 co-inhibition [145].

Additionally, the mTORC2 substrate SGK1 can replace AKT in response to PI3K/AKT inhibition, leading to the activation of shared AKT substrates that mediate oncogenic cellular processes such as cell growth, survival metabolism, and migration [145,267] (Figure 2B). In *PIK3CA* mutant breast cancer cells, PDK1-SGK1 signaling has been shown to sustain AKT-independent mTORC1 activation to promote resistance to the p110α-isoform-specific PI3K inhibitor BLY719, and PDK1 or SGK1 blockade can restore BYL719 sensitivity [145]. Furthermore, elevated SGK1 can predict for AKT inhibitor resistance in breast cancer cells [267]. Interestingly, Class I PI3K and AKT inhibition has also been shown to increase PI3K Class III hVsp34-SGK3 signaling in breast cancer cells, which can substitute for AKT by phosphorylating TSC2 to activate mTORC1 [148]. Whether SGK1/3 shares AKT’s ability to phosphorylate and activate RAF is currently unknown.

#### 3.1.2. Co-targeting RAS/MAPK and PI3K-AKT-mTOR Signaling in Prostate Cancer

Co-activation of the RAS/MAPK and PI3K-AKT-mTOR signaling pathways occurs frequently in human malignancies including prostate cancer, thus considerable research has been devoted to establishing how these two oncogenic cascades interact [101,256,257,268,269,270]. Nearly all metastatic prostate cancer patients are reported to show deregulation of both cascades [43]. To model this in vivo, genetically engineered mouse models of prostate cancer with prostate specific *Pten* homozygous deletion harboring either a *KRas*^G12D^ activating mutation or oncogenic *BRaf*^V600E^ with NK3 Homeobox 1 (*Nkx3.1*) depletion, promotes rapid tumor growth and metastatic progression relative to the single mutants [101,268,269]. To our knowledge, these tumor models were the first immunocompetent transgenic mouse models of prostate adenocarcinoma to display reproducible metastatic disease.

Taken together, these findings indicate that PI3K-AKT-mTOR and RAS/MAPK signaling synergize to promote prostate cancer growth and metastatic progression, and given the frequency of co-activation of these cascades in the clinic, this provides a clear justification for exploring the combination of PI3K-AKT-mTOR and RAS/MAPK pathway inhibitors in patients with advanced prostate cancer. This notion is further supported by the fact that MEK inhibition is associated with elevated PI3K-AKT-mTOR signaling in mammalian cancer cells, including prostate cancer cells [271,272]. Preclinical studies have also shown that co-inhibition of MEK and mTORC1 can significantly reduce tumor burden relative to monotherapy in a mouse model of prostate cancer driven by simultaneous heterozygous deletion of *Nkx3.1* and *Pten* [256], and can inhibit cell growth and increase cytotoxicity in the castration-resistant CWR22Rv1 human prostate cancer cell line [272]. However, MEK inhibition alone is reported to be sufficient to suppress the metastatic spread of *Pten*-deleted and *KRas* activated stem/progenitor murine prostate cancer cells orthotopically transplanted in vivo, similarly to combined mTORC1 and MEK inhibition [101]. This highlights the need to improve our molecular understanding of how these cascades interact during disease progression and in the presence of different genetic drivers to aid the stratification of patients that will benefit from (a) PI3K-AKT-mTOR inhibition, (b) MEK inhibition or (c) combined PI3K-AKT-mTOR and RAS/MAPK blockade.

Several prostate cancer clinical trials have been designed to investigate the therapeutic efficacy of targeting MEK (e.g.,MEK1/2 inhibitor trametinib, ClinicalTrials.gov identifiers: NCT02881242 and NCT01990196) or the PI3K-AKT-mTOR cascade (e.g., pan-AKT inhibitors including ipatasertib and capivasertib, ClinicalTrials.gov identifiers: NCT01485861/NCT03673787 and NCT02525068/ NCT02121639 respectively) [134,273]. Metformin (an oral type 2 anti-diabetic drug) is also currently being investigated in prostate cancer within the STAMPEDE trial [274]. Metformin targets the mitochondrial respiratory chain complex I, leading to reduced mitochondrial ATP production that causes cellular energy crisis with subsequent AMPK activation and mTORC1 inhibition [275]. Metformin has also been shown to inhibit MEK/ERK in response to growth factors, contrasting mTORC1 inhibitor treatment with rapamycin that increases MAPK signaling [276]. 

Although not currently specific to patients with prostate cancer, clinical trials exploring co-inhibition of the PI3K-AKT-mTOR and MAPK cascades to treat various advanced solid cancers have also been developed (e.g., ClinicalTrials.gov identifiers: NCT01390818, NCT01347866, and NCT02583542), although response rates appear to be low and are linked to *RAS* and *RAF* mutations [277]. For example, a recent Phase Ib study of combination therapy with the MEK1/2 inhibitor binimetinib (Mektovi) and the pan-PI3K inhibitor Buparlisib (BKM120) in advanced solid tumors reported promising efficacy in patients with advanced ovarian cancer with *RAS*/*RAF* genetic alterations, however continuous dosing resulted in intolerable toxicities and an intermittent schedule is suggested for future trials [278]. Additionally, the MATCH screening trial (targeted therapy directed by genetic testing in treating patients with advanced refractory solid tumors, lymphomas, or multiple myeloma, ClinicalTrials.gov identifier: NCT02465060) will investigate the efficacy of MEK and PI3K inhibitors as monotherapies in patients with progressive disease that carries a genetic alteration in either the RAS/MAPK or the PI3K-AKT-mTOR pathways respectively.

### 3.2. PI3K-AKT-mTOR and AR Signaling Crosstalk

AR signaling regulates cell growth, differentiation, migration and survival, and plays a critical role as a transcriptional regulator during prostate development, normal prostate tissue homeostasis, and prostate cancer [279,280,281]. AR is a steroid nuclear receptor that transmits androgen signals such as testosterone (T), or its more potent metabolite dihydrotestosterone (DHT), to regulate gene expression and coordinate cellular responses. T is derived from cholesterol through a cascade of biochemical reactions involving four enzymes: cytochrome P450 side-chain cleavage enzyme (P450scc), cytochrome P450 17α-hydroxylase/17,20-lyase (CYP17A1), 3β-hydroxysteroid dehydrogenase (3β-HSD) and 17β-hydroxysteroid dehydrogenase (17β-HSD) [282]. The conversion of cholesterol to pregnenolone is catalyzed by P450scc, and its subsequent conversion to progesterone is catalyzed by 3β-HSD. Pregnenolone and progesterone can be converted by CYP17A1 to 17-OH-pregnenolone and 17-OH-progesterone and subsequently to dehydroepiandrosterone (DHEA) and androstenedione (AD or A4). DHEA and AD may then be converted to androstenediol and T by 17β-HSD [282]. T synthesis and secretion predominantly occurs in the Leydig cells of the testes, and is stimulated by pituitary-derived luteinizing hormone (LH), which is secreted in response to hypothalamus-derived LHRH, (also known as gonadotrophin-releasing hormone, GnRH) [281,283]. In the prostate, 5-alpha-reductase converts T to DHT [281,283]. In addition, androgens can also be produced by the adrenal glands and in some instances by prostate tumor cells [284], which may contribute to prostate cancer growth post-orchiectomy [285].

In the absence of androgens, AR forms a cytoplasmic complex with chaperones (e.g., HSP90 and HSP70). Androgen binding displaces the chaperones and triggers a conformational change in AR, which enables AR homodimerization and nuclear translocation (Figure 1) [279,281,286]. Nuclear AR homodimers regulate the transcription of androgen-regulated genes (e.g., *SLC43A1*, *FKBP5, CAMKK2, NKX3.1* and *KLK3*) by directly binding to an androgen responsive element (ARE) in the promotor/enhancer region of target genes [279,286]. However, growth factors (e.g., EGF and IGF-1), cytokines (e.g., IL6) and intracellular signaling kinases (e.g., AKT and SRC) can also independently stimulate AR dependent transcriptional activity when androgen levels are low, which can facilitate therapeutic resistance to androgen/AR blockade [12,281,287]. In addition to regulating gene transcription, AR can also mediate a number of intracellular signaling pathways through direct protein–protein interactions within the cytoplasm (known as non-genomic AR signaling) [12,281]. For instance, AR is reported to activate SRC family kinases, PKC, RAS, ERK, PI3K, and AKT [12,288].

Aberrant AR signaling is a common feature of prostate cancer [289], with up to 56% of primary cases and 100% of metastatic cases reported to carry genetic alterations within key AR pathway components [43]. While the majority of men with prostate cancer initially respond to androgen/AR-directed therapy, they inevitably develop castration-resistant prostate cancer (CRPC), as malignant cells develop therapeutic resistance [5,290]. Several inherent and acquired resistance mechanisms have been identified, including AR genetic alterations (e.g., activating mutations, gene amplification, androgen-independent constitutively active splice variants, AR loss), augmented androgen biosynthesis, adrenal androgens, AR-bypass signaling (e.g., glucocorticoid receptor (GR) regulation of shared AR target genes), trans-differentiation to neuroendocrine prostate cancer and ligand-independent activation via crosstalk with another signaling cascade, such as the PI3K-AKT-mTOR pathway [12,283].

**Figure 3:**
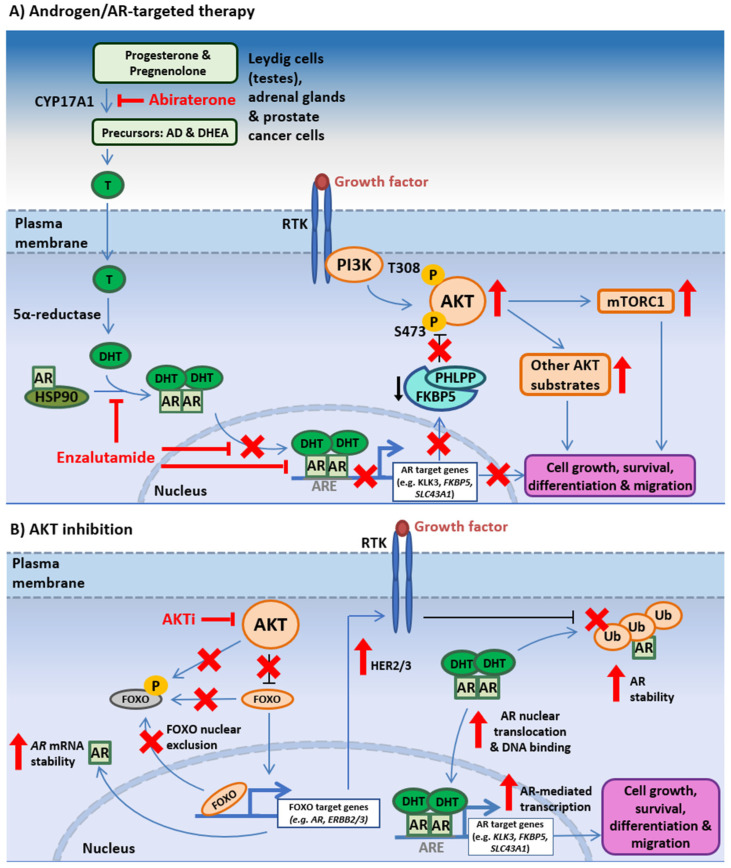
PI3K-AKT-mTOR and AR signaling crosstalk facilitates resistance to androgen/AR and AKT-directed monotherapy. Schematics depict reported model mechanisms for therapeutic resistance to (**A**) androgen/AR-directed therapy, which leads to increased AKT activation, and (**B**) AKT inhibition. AD, androstenedione; CYP17A1, cytochrome P450 17A1; DHEA, dehydroepiandrosterone; Ub, ubiquitination event.

The PI3K/AKT/mTOR and AR pathways have been shown to cross-regulate through several reciprocal inhibitory loops [11,12,24] (Figure 3). Consequently, the PI3K-AKT-mTOR pathway can be inadvertently activated in response to androgen/AR-directed therapies, and vice versa PI3K-AKT-mTOR pathway inhibition can augment AR signaling, leading to therapeutic resistance. Human patient samples (both primary tumor and bone metastases), human prostate cancer cell lines and transgenic mouse models of prostate cancer have consistently demonstrated that AKT-mTOR signaling is increased in response to androgen/AR-directed blockade [11,13,24,291,292,293]. Mechanistically, it is reported that inhibiting AR signaling reduces expression of the AR target gene FK506-binding protein-5 (*FKBP5*), which leads to PHLPP destabilization and reduced PHLPP-mediated dephosphorylation of AKT at Ser473 to promote AKT signaling [11,24] (Figure 3A). Thus, compensatory activation of the PI3K-AKT-mTOR pathway in response to androgen/AR pathway inhibition can facilitate CRPC growth.

Conversely, PI3K-AKT-mTOR pathway inhibition is associated with augmented AR signaling that can contribute to drug resistance and promote prostate cancer progression [11,293,294,295]. Carver and colleagues showed that PI3K/mTOR inhibition activates AR signaling in human xenograft and transgenic mouse models of prostate cancer, and that co-treatment with the PI3K/mTOR inhibitor BEZ235 and the antiandrogen MDV3100 (enzalutamide) significantly reduced tumor burden relative to monotherapy [11]. In corroboration, resistance to the AKT inhibitor capivasertib (AZD5363) in LNCaP prostate cancer xenografts is also associated with elevated AR signaling, and combining AZD5363 treatment with the antiandrogen bicalutamide prolonged disease stabilization [295]. Furthermore, mTOR and EGFR co-inhibition with everolimus and gefitinib has shown limited sensitivity in patients owing to enhanced AR activity and PSA levels [293], providing further rationale for combining AR and PI3K-AKT-mTOR blockade to treat prostate cancer.

Several distinct molecular mechanisms have been identified that underpin AR reactivation upon AKT inhibition. Notably, AKT inhibition can prevent AKT-mediated nuclear exclusion of FOXOs, which can lead to augmented transcription of FOXO-target genes such as RTKs (e.g., *ERBB2/3* encoding HER2/3) (Figure 3B) [11,262,296]. HER2/3 activity has been shown to promote AR signaling by protecting AR from ubiquitination and proteasomal degradation, and by enhancing AR binding to ARE target sequences and stimulating AR transcriptional activity [297,298,299] (Figure 3B). Nonetheless, the role of FOXO-dependent signaling in PI3K-AKT-mTOR and AR pathway crosstalk is complex. Although FOXO transcription factors upregulate the expression of RTKs [262] causing a subsequent increase in AR signaling [298], the ectopic expression of FOXO1 conversely dampens AR activity, which is further exacerbated when FOXO1 is co-transfected with the AR coregulator HDAC3 [300]. 

PTEN loss has also been shown to downregulate AR signaling via the upregulation of several factors that inhibit AR signaling through histone modification mechanisms such as early growth response 1 (EGR1), transcription factor AP-1 (c-JUN), and the catalytic subunit of polycomb repressive complex 2 enhance of zeste homolog 2 (EZH2) (Figure 1) [24]. PTEN protein-phosphatase activity has also been shown to protect the tumor suppressor NK3 homeobox 1 (NKX3.1) from degradation, which can derail the AR transcriptional network [301]. Of note, NKX3.1 and AR can cross-regulate [302], and enforced NKX3.1 expression can suppress *Pten*-deleted prostate tumorigenesis in transgenic mice [303]. 

Despite the antagonistic crosstalk between AR and AKT (Figure 3), AR signaling can boost mTORC1 activation through an AR-dependent increase in amino acid transport during tumorigenesis [304]. AR mediates expression of L-type amino acid transporters (e.g., LAT3 encoded by *SLC43A1*) to maintain sufficient levels of leucine needed for mTORC1 signaling and cell growth (Figure 1 and Figure 3) [304]. Moreover, LAT1 and LAT3 transport inhibition is sufficient to decrease cell growth and mTORC1 signaling in prostate cancer cells in vitro [304]. Recent in vitro data have also revealed that mTOR can directly interact with AR in the nucleus of prostate cancer cells to promote metabolic rewiring, and high levels of nuclear mTOR correlate with poor prognosis in patients with prostate cancer [293]. Additionally, AKT has been shown to directly bind and phosphorylate AR when T levels are low, although the functional significance of this event remains to be determined [16,305,306].

The reciprocal feedback loop between AR and PI3K-AKT-mTOR signaling may also be perturbed by Speckle-type BTB/POZ protein (*SPOP*) loss of function mutations that lead to the stabilization of the SPOP substrate SRC3 (e.g., p.F133V), which consequentially increases PI3K activity [307]. SPOP is an adaptor protein of the Cullin 3 family E3 ligases that can target SRC3 for ubiquitin-mediated degradation and a known tumor suppressor [12,308,309,310]. In prostate cancer, *SPOP* is frequently mutated (9–11% incidence) [45,46]. Remarkably, *SPOP* mutation can also stabilize AR and potentiate AR signaling whilst the PI3K-AKT-mTOR signaling pathway is activated, allowing coordinated and cooperative signaling that drives tumorigenic growth [307]. Conversely, wildtype SPOP can trigger E3 ligase mediated degradation of AR via hinge domain binding when androgens levels are low [311]. Furthermore, AR has been shown to positively regulate the PI3K-AKT-mTOR pathway via a direct interaction with the SH2 domain of the Class IA PI3K regulatory subunit p85α, which has been shown to activate the PI3K-AKT-mTOR cascade [312], further highlighting the complexity of the interactions between these two cascades.

Taken together, these findings support the rationale for combining pharmacological inhibition of the AR and PI3K-AKT-mTOR cascades to treat prostate cancer in the clinic, and highlight the need for further work to delineate the molecular mechanisms underpinning crosstalk between these two oncogenic cascades. Importantly, clinical trials exploring co-targeting AR and PI3K-AKT-mTOR signaling are beginning to show promise. A randomized Phase Ib/II study combining the pan-AKT inhibitor ipatasertib with abiraterone in mCRPC patients has reported ipatasertib + abiraterone prolongs radiographic progression-free survival (rPFS), improves overall survival and extends time to PSA progression compared to abiraterone alone, particularly in patients with PTEN loss (ClinicalTrials.gov identifier: NCT01485861) [134]. This study also reports that the adverse effects common to PI3K-AKT-mTOR blockade (e.g., hyperglycemia) were generally clinically manageable [134]. A Phase I dose escalation study combining enzalutamide and capivasertib to treat mCRPC has also recently reported 3/16 patients responded (ClinicalTrials.gov identifier: NCT02525068) [313]. In this study, patients who met the response criteria had *PTEN* loss or *AKT* activating mutations, low/absent AR-V7 protein levels and elevated p-ERK [313]. Nevertheless, several additional clinical trials investigating the combination of AR and PI3K-AKT-mTOR blockade in men with mCRPC did not demonstrate a therapeutic benefit and were associated with poor tolerability (ClinicalTrials.gov identifiers: NCT01385293, NCT01634061 and NCT01717898) [314,315,316]. Interestingly, D’Abronzo and colleagues also recently showed that eIF4E phosphorylation at residue Ser209 in human CRPC cell lines pre-treated with the antiandrogen bicalutamide underpins resistance to subsequent combination therapy with bicalutamide + rapamycin treatment. Remarkably, suppression of eIF4E phosphorylation by MNK1/2 (MAP kinase interacting serine/threonine kinase1/2) or ERK1/2 inhibition was shown to sensitize bicalutamide pre-treated CRPC cells to combined anti-androgen and mTORC1 blockade [317], presenting a novel avenue for overcoming therapeutic resistance. Thus, despite some promising results, it is evident that further investigation into the molecular mechanisms underpinning AR and PI3K-AKT-mTOR pathway crosstalk in prostate cancer is required to improve patient stratification and to discover new therapeutic approaches and predictive biomarkers that can inform future clinical trial design.

### 3.3. PI3K-AKT-mTOR and WNT Signaling Interactions

The WNT family is an evolutionarily conserved group of proteins essential for growth control, organ development, tissue homeostasis and stem cell renewal in multiple organs, and is crucial for normal prostate development [318,319]. WNT signaling is potentiated by secreted WNT ligands (a family of 19 lipoglycoproteins) that bind extracellularly to transmembrane frizzled receptors (FZD1-10) and their co-receptors, such as low-density lipoprotein receptors (e.g., LRP5 and LRP6), tyrosine protein-kinases (e.g., receptor tyrosine kinase–like orphan receptor-1 and -2, ROR1/2), and tyrosine kinase-related receptors (e.g., receptor-like tyrosine kinase, RYK, protein tyrosine kinase 7, PTK7, and muscle specific kinase, MuSK) [318,319,320]. The WNT signal is transduced intracellularly via dishevelled (DVL), which subsequently activates either β-catenin-dependent/canonical WNT signaling or β-catenin-independent/non-canonical WNT signaling [318,319,320]. In the absence of a canonical WNT ligand, cytosolic β-catenin levels are maintained at a low level via the β-catenin destruction complex that contains the scaffold protein AXIN, the tumor suppressor adenomatous polyposis coli (APC), GSK3β and casein kinase 1 (CK1). The β-catenin destruction complex phosphorylates β-catenin, leading to its ubiquitylation and proteasomal degradation [321]. Canonical WNT signals disrupt the β-catenin destruction complex, resulting in β-catenin stabilization and accumulation, nuclear translocation and interaction with TCF/LEF transcription factors to upregulate WNT target genes such as *MYC* and *AXIN2* [321] (Figure 1). Non-canonical WNT signaling involves WNT-mediated activation of RhoA/ROCK and RAC/JNK/NFAT signaling (planar cell polarity pathway), or phospholipase C (PLC) activation and the accumulation of intracellular Ca^2+^ that stimulates calmodulin-dependent kinase II (CamKII), calcineurin and protein kinase C (PKC) signaling (WNT/Ca^2+^ pathway) [320].

Activation of both the canonical and non-canonical WNT cascades has been reported in localized and advanced prostate cancer, and oncogenic deregulation of core WNT pathway components frequently occurs in primary and metastatic prostate cancer (up to 6% and 19% incidence respectively [45]), primarily via *APC* deep deletion/truncating mutations and *CTNNB1*/β-catenin activating mutations [45,46,320,322,323]. Furthermore, the WNT/β-catenin pathway is strongly linked to androgen/AR-directed therapy and chemotherapy resistance [324,325,326,327], thus WNT signaling presents an attractive therapeutic target for advanced prostate cancer. In addition, *AR* is in fact a WNT/β-catenin target gene, and AR and β-catenin can directly interact and co-localize in the nucleus to mediate transcriptional activity of AR-regulated genes [328,329,330] (Figure 1). WNT and AR signaling cascades have also been shown to reciprocally inhibit each other in murine prostate cancer [331].

Mouse models have been instrumental in determining the role of WNT signaling in prostate cancer, and we and others have shown that constitutive activation of β-catenin or *Apc* bi-allelic deletion predisposes to prostate adenocarcinoma in mice [269,331,332,333]. Moreover, β-catenin activation can cooperate with *Pten* heterozygous or homozygous deletion to promote prostate cancer progression, CRPC transition and metastatic potential [269,331,332,333], indicating a synergistic relationship exists between the PI3K-AKT-mTOR and WNT cascades.

**Figure 4:**
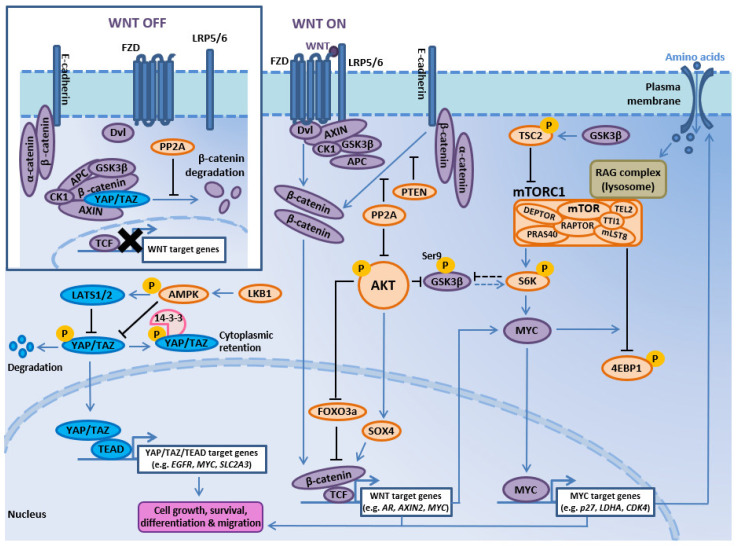
PI3K-AKT-mTOR and WNT signaling crosstalk. Upon ligand binding (WNT ON), the destruction complex is recruited to the plasma membrane leading to β-catenin accumulation in both the cytoplasm and nucleus, where it activates gene expression through TCF binding. Insert illustrates WNT signaling in the absence of WNT ligand (WNT OFF). The interplay between PI3K-AKT-mTOR and WNT may occur through shared pathway components (e.g., GSK3β, PTEN, PP2A) and/or the joint regulation of transcription factors such as MYC, FOXO3a, SOX4 or YAP/TAZ. CDK4, cyclin-dependent kinase 4; LATS1/2, l-type amino acid transporter 1/2; LDHA, l-lactate dehydrogenase A chain; SLC2A3, solute carrier family 2, facilitated glucose transporter member 3 (encoding GLUT3, glucose transporter 3); TAZ, transcriptional co-activator with PDZ-binding motif; TEAD, transcriptional enhanced associate domain transcription factor; YAP, Yes-associated protein.

Several molecular mechanisms that permit cross-regulation of the PI3K-AKT-mTOR and WNT signaling cascades have emerged in the literature, which could influence prostate cancer growth and resistance to anti-androgens and/or PI3K-AKT-mTOR pathway inhibitors [320,322,323,334,335] (Figure 4). While canonical WNT signaling mediates cellular β-catenin levels, the level of active β-catenin (unphosphorylated at residues Ser37 and Thr41) in melanoma, breast and prostate cancer cells is reported to be regulated by the PI3K-AKT-mTOR cascade, in a process that is dependent on PP2A activity [335]. PP2A is known to negatively regulate AKT, however it is currently speculated that this phosphatase may also directly dephosphorylate and activate β-catenin [335]. Additionally, the PI3K-AKT-mTOR pathway has also been shown to mediate β-catenin localization [335], and several transcription factors that directly interact with β-catenin are co-regulated by the PI3K-AKT-mTOR pathway, such as FOXO3a [336] and SOX4 [337]. In prostate cancer cells, FOXO3a has been shown to suppress β-catenin transcriptional activity and can be inhibited by AKT [336], whereas SOX4 is a positive regulator of canonical WNT and is stimulated by AKT [338] (Figure 4).

In addition to transmitting canonical WNT signals, β-catenin also form adherens junctions with α-catenin and E-cadherin at cell–cell junctions to maintain tissue architecture and facilitate cell–cell signaling (Figure 4). PTEN has also been shown to modulate β-catenin nuclear localization and transcriptional activity through caveolin-1 (CAV1)-dependent dissociation of β-catenin from E-cadherin at the membrane independently of PI3K-AKT-GSK3β signaling, leading to increased tumor formation and metastatic progression in melanoma [339].

Crosstalk between the PI3K-AKT-mTOR and WNT/β-catenin signaling pathways is also mediated via GSK3β and the TSC1:TSC2 complex [167] (Figure 4). GSK3β play a critical role in both cascades, serving as a core member of the β-catenin destruction complex that helps to maintain low level of cytosolic/nuclear β-catenin in the absence of WNT signal [340], and as a direct substrate of AKT [167]. AKT inactivates GSK3β by phosphorylating residue Ser9 [167]. GSK3β can also phosphorylate and activate TSC2 resulting in inhibition of mTOR activity [167], and can restrict cellular growth by suppressing glucose uptake via TSC2 and mTOR [341]. Active WNT signaling inhibits GSK3β, abrogates the suppression of mTOR and stimulates phosphorylation of S6K, S6, and eukaryotic translation initiation factor 4E binding protein 1 (4EBP1) [167]. Interestingly, in the absence of TSC1 or TSC2, S6K has also been shown to inactivate GSK3β by directly phosphorylating residue Ser9 and active GSK3β has also been shown to phosphorylate/activate S6K, adding further complexity to GSK3β signaling between the WNT and PI3K-AKT-mTOR cascades [342]. However, previous work has also indicated that GSK3β does not mediate crosstalk between the PI3K-AKT-mTOR and WNT/β-catenin pathways [343,344], raising the possibility that GSK3β function is context/tissue dependent. Of note, WNT ligands can also activate mTOR through MYC-dependent suppression of TSC2 [345].

PI3K-AKT-mTOR and WNT signaling may also interact through Hippo signaling. Hippo signaling is tightly intertwined with cell size regulation and nutrient sensing through LKB1-AMPK, TSC1:TSC2 and mTOR [346,347], and the Hippo pathway signaling proteins yes-associated protein (YAP) and transcriptional co-activator with PDZ-binding motif (TAZ) are integral parts of both canonical and non-canonical WNT signaling [348,349]. YAP and TAZ are members of the β-catenin destruction complex, and in the presence of WNT signals, they dissociate from the complex and translocate to the nucleus to activate downstream targets [348] (Figure 4). AMPK activation has also been shown to negatively regulate YAP/TAZ activity [347].

Non-canonical WNT signaling has also been shown to activate the PI3K-AKT-mTOR pathway. For example, WNT/FZD7-dependent dissociation of Gβγ from Gαi enhances PI3K-AKT signaling and increases tumor cell invasive potential [350]. ROR1 can also activate PI3K-AKT signaling in response to trans-phosphorylation by tyrosine kinases, such as MET and SRC [351]. In addition, WNT receptor Frizzled2 (FZD2) can drive epithelial-to-mesenchymal transition (EMT) and cell migration through activation of Fyn [352], and activated Fyn kinase activity has been shown to suppress the AMPK-LKB1 signaling axis by blocking LKB1 redistribution into the cytoplasm [353].

Interestingly, the PI3K-AKT-mTOR, WNT, MAPK and AR signaling cascades all converge to regulate the transcription factor MYC, which is frequently amplified in prostate cancer/mCRPC [44,45,354]. While *MYC* is a WNT/β-catenin target gene [355], PI3K-AKT-mTOR signaling can mediate *MYC* mRNA stability, translation and protein stability [356,357,358,359,360]. AR signaling has also been shown to stimulate MYC in AR-driven prostate cancer, while in normal prostate tissue AR silences *MYC* to maintain normal homeostasis [361,362]. However, MYC is also reported to antagonize AR transcriptional activity in prostate cancer [363]. MYC upregulation is frequently observed in prostate cancer, and although targeting MYC remains a clinical challenge, preclinical studies have emphasized the potential efficacy of MYC blockade for patients with late-stage prostate cancer [364].

WNT inhibitors are beginning to enter clinical trials, including small-molecule inhibitors to the enzyme porcupine that block WNT ligand secretion, such as WNT974 (LGK974) [320,322]. Preliminary data from a WNT974 phase 1 clinical trial (NCT01351103) for a small range of human malignancies (excluding prostate cancer) report a manageable safety profile and suppression of canonical WNT/β-catenin target gene *AXIN2* [320], and recent preclinical studies have indicated that WNT974 treatment is efficacious against prostate cancer [331,365]. β-catenin has also been reported to facilitate resistance to PI3K and AKT inhibition in colon cancer [334]. Thus, further work exploring the therapeutic benefit of targeting the WNT pathway in prostate cancer is warranted.

## 4. Conclusions

In summary, the PI3K-AKT-mTOR cascade is frequently activated in prostate cancer, and genomic profiling has revealed that oncogenic genetic alterations occur within a diverse array of PI3K-AKT-mTOR pathway components. Significant research efforts have been devoted to delineating the mode of action of several of these aberrations (e.g., *PTEN* deletion and *PIK3CA* activating mutation), however our molecular understanding of how these events differentially mediate cell signaling programs is limited, and several genetic alterations remain to be studied functionally. Future work to gain novel insight into the functional consequence of these genetic alterations in prostate cancer is necessary to (a) identify passenger vs. driver alterations, (b) establish the ability of individual aberrations to synergize with additional oncogenic events, and (c) discover their mode of action during tumor growth, metastasis and therapeutic resistance. In conjunction with genomic and transcriptomic data, establishing the frequency and impact of post-transcriptional modifications and epigenetic events within core PI3K-AKT-mTOR pathway components during prostate tumorigenesis and disease progression/recurrence is also crucial, as these components are regulated at multiple levels and genomic/transcriptomic data do not consistently equate with protein activity.

Targeting the PI3K-AKT-mTOR pathway in prostate cancer remains a key clinical challenge. Therapeutic resistance emerges owing to various feedback/feedforward loops and redundancy mechanisms that prevent complete suppression of the pathway and cause compensatory augmentation of interacting signaling pathways, thus rationalizing the exploration of combination therapies. Encouragingly, clinical trials are beginning to report therapeutic efficacy when combining PI3K-AKT-mTOR and androgen/AR-directed therapies, particularly in patients with mCRPC that display PTEN loss. However, the mechanism of resistance to PI3K-AKT-mTOR pathway-targeted therapies is likely to vary dramatically between patients and within individual tumors owing to several factors. These include the activity status of the pathway components, the extent of intratumoral heterogeneity, the mode and concentration of upstream stimuli, the genetic alterations present and the composition of the tumor microenvironment. Furthermore, our ability to successfully translate preclinical findings to the clinic is currently hampered by the limited number of prostate cancer preclinical models available, which do not fully cover the broad range of prostate cancer subtypes or disease heterogeneity seen in the clinic. Accordingly, to discover newtherapeutic approaches that increase patient response rates and overall survival, further delineation of the complex signaling network that exists within the PI3K-AKT-mTOR pathway and the interacting MAPK, AR, and WNT pathways is needed, together with the development of a wider range of preclinical models that better recapitulate the clinic and a deeper understanding of the molecular biology underpinning prostate cancer disease subtypes and tissue heterogeneity.

## Figures and Tables

**Table 1:** Frequency of common genetic alterations in PI3K-AKT-mTOR pathway genes in prostate cancer.

Common Types of Genetic Alterations in PI3K-AKT-mTOR Pathway Genes	Frequency in Prostate Cancer ^1^
*PTEN* deletion/mutation	16.4–32.0%
*DEPTOR* amplification	5.1–21.4%
*SGK* mutation/amplification	5.6–20.5% (*SGK3*)
	0.2–2.7% (*SGK1*)
*FOXO* deletion	0.0–15.2% (*FOXO1*)
	4.5–13.4% (*FOXO3*)
*MAP3K7* deletion	5.9–14.8%
*RRAGD* deletion	6.5–14.4%
*SESN1* mutation/deletion	5.4–13.6%
*PIK3CA* mutation/amplification	5.5–11.5%
*PIK3C2B* mutation/amplification	1.4–11.5%
*PDPK1* amplification	0–8.1%

^1^ Data sourced from the Memorial Sloan Kettering Cancer Centre/Dana-Farber Cancer Institute (MSKCC/DFCI) (*n* = 1013) [45] and The Cancer Genome Atlas (TCGA), Firehose Legacy (*n* = 492) prostate adenocarcinoma datasets, and the metastatic prostate adenocarcinoma Stand Up To Cancer & Prostate Cancer Foundation International Dream Team (SU2C-PCF IDT) dataset (*n* = 444) [46] using cBioPortal [47,48] (Tables S1–S3). Only samples with mutation and copy number alteration (CNA) data were analyzed. The percentage frequency range for each genetic alteration listed reflects the entire patient population across all the three datasets, irrespective of the disease stage or subtype.

## Supplementary Materials

Supplementary Materials can be found at https://www.mdpi.com/1422-0067/21/12/4507/s1.

## Abbreviations

3β-HSD	3β hydroxysteroid dehydrogenase
4E-BP1	Eukaryotic translation initiation factor 4E binding protein 1
ADT	Androgen deprivation therapy
AD	Androstenedione
AGC	cAMP-dependent, cGMP-dependent and protein kinase C
AKTi	AKT inhibitor
AMP	Adenosine monophosphate
AMPK	5′ AMP-activated protein kinase
APC	Adenomatous polyposis coli
AR	Androgen receptor
ARE	Androgen responsive element
ARF1	Adenosine ribosylation factor 1
ATG14	Autophagy related 14 homolog
ATP	Adenosine triphosphate
AXIN	Axis inhibitor protein
BAD	Bcl-2-associated death promoter
BMK-1	Big mitogen-activated protein kinase-1
CaMKII	Calmodulin-dependent kinase 2
CAMKKβ	Ca(2+)/calmodulin-dependent protein kinase kinase β
CAV1	Caveolin-1
CDK4	Cyclin-dependent kinase 4
c-JUN	Transcription factor AP-1
CK1	Casein kinase 1
CAN	Copy number alteration
CREB	cAMP response element-binding protein
CRPC	Castrate resistant prostate cancer
CYP17A1	Cytochrome P450 17A1
DEPTOR	Dishevelled, EGL-10 and pleckstrin (DEP) domain-containing mTOR-interacting protein
DFCI	Dana-Farber Cancer Institute
DHEA	Dehydroepiandrosterone
DHTDVL	DihydrotestosteroneDisheveled
EIF4E	Eukaryotic translation initiation factor 4E
EGF	Epidermal growth factor
EGFR	Epidermal growth factor receptor
EGR1	Early growth response 1
EMT	Epithelial-to-mesenchymal transition
ERG1	ETS-related Gene 1
ERK1	Mitogen-activated protein kinase 3
ERK2	Mitogen-activated protein kinase 1
EZH2	Enhancer of zeste homolog 2
FKBP5	FK506 binding protein 5
FOXO	Forkhead box protein O
FGFR	Fibroblast growth factor receptor
FZD	Frizzled family receptor
GAB1	GRB2-associated binder-1
GDP	Guanosine diphosphate
GLUT3	Glucose transporter 3
GnRH	Gonadotrophin-releasing hormone
GPCR	G-protein coupled receptor
GR	Glucocorticoid receptor
GRB2	Growth factor receptor-bound protein 2
GRB10	Growth factor binding protein 10
GSK3β	Glycogen synthase kinase 3 beta
GTP	Guanosine triphosphate
HDAC3	Histone deacetylase 3
HER2/3	Human epidermal growth factor receptor 2/3
HSD3B/17B3	Hydroxysteroid dehydrogenase 3B/17B3
HSP70/90	Heat shock protein 70/90
IGF	Insulin growth factor
IGF-1	Insulin growth factor 1
IHC	Immunohistochemistry
IL6	Interleukin 6
INPP4B	Inositol polyphosphate 4-phosphatase type II
INPP5D	Inositol polyphosphate-5-phosphatase D
INPP5J	Inositol polyphosphate-5-phosphatase J
INPPL1	Inositol polyphosphate phosphatase like 1
IRS	Insulin receptor substrate
IRS1	Insulin receptor substrate protein 1
KLK3	Kallikrein related peptidase 3
LAT1/2/3	L-type amino acid transporter 1/2/3
LDHA	L-lactate dehydrogenase A chain
LEF	Lymphoid enhancer binding factor
LH	Luteinizing hormone
LHRH	Luteinizing hormone-releasing hormone
LKB1	Liver kinase B1
LRP5/6	Low-density lipoprotein receptor-related proteins 5 and 6
MAPK	Mitogen-activated protein kinase
MAP3K7	Mitogen-activated protein kinase kinase kinase 7
mCRPC	Metastatic castrate resistant prostate cancer
MDM2	Mouse double minute 2 homolog
MEK	Mitogen-activated protein kinase kinase
MSKCC	Memorial Sloan Kettering Cancer Centre
mLST8	MTOR associated protein LST8 homolog
MMTV-PyMT	Mouse mammary tumor virus-polyoma middle tumor-antigen
MNK1	MAP kinase interacting serine/threonine kinase 1
MNK2	MAP kinase interacting serine/threonine kinase 2
mSIN1	Mitogen-activated protein kinase associated protein 1 (MAPKAP1)
mTOR	Mammalian target of rapamycin
mTORC1	Mammalian target of rapamycin complex 1
mTORC1i	mTORC1 inhibitor
mTORC2	Mammalian target of rapamycin complex 2
MuSK	Muscle specific kinase
NF-κB	Nuclear factor kappa light chain enhancer of activated B cells
NKX3.1	NK3 Homeobox 1
NSCLC	Non-small-cell lung carcinoma
P	Phosphorylation event
PDK1	Phosphoinositide-dependent kinase 1
PGC-1α	Peroxisome proliferator-activated receptor gamma coactivator 1-alpha
PHLPP	PH domain leucine-rich repeat protein phosphatase
PHLPP1	PH domain and leucine rich repeat protein phosphatase 1
PHLPP2	PH domain and leucine rich repeat protein phosphatase 2
PI	Phosphatidylinositol
PI3K	Phosphoinositide 3-kinase
PI(3)P	Phosphatidylinositol 3-phosphate
PI(3,4)P2	Phosphatidylinositol 3,4-bisphosphate
PIN	Prostate intra-epithelial neoplasia
PIP2	Phosphatidylinositol 4,5-bisphosphate
PIP3	Phosphatidylinositol 3,4,5-trisphosphate
PIPP	Proline-rich inositol polyphosphate 5-phosphatase
PKB	Protein kinase B, AKT
PKC	Protein kinase
PKCα	Protein kinase C alpha
PLC	Phospholipase C
PP2A	Protein phosphatase 2A
PPP2CA	Protein phosphatase 2 catalytic subunit Alpha
PRAS40	Proline-rich AKT substrate of 40 kDa
PROTOR1	Protein observed with Rictor-1
PROTOR2	Protein observed with Rictor-2
PSA	Prostate specific antigen
PTEN	Phosphatase and tensin homologue deleted on chromosome 10
PTK7	Protein tyrosine kinase 7
RAF	Rapidly accelerated fibrosarcoma
RAG	Recombination activating genes
RAPTOR	Regulatory-associated protein of mTOR
RBD	Ras-binding domain
RHEB	Ras homolog enriched in brain
RICTOR	Rapamycin-insensitive companion of mTOR
ROR1/2	Receptor tyrosine kinase–like orphan receptor-1 and -2
RPS6	Ribosomal protein S6
rPFS	Radiographic progression-free survival
RSK	90 kDa Ribosomal S6 kinase
RTK	Tyrosine kinase receptor
RYK	Receptor-like tyrosine kinase
S6K	Ribosomal protein S6 kinase/p70 ribosomal S6 kinase
SESN1	Sestrin 1
SGK1	Serum and glucocorticoid regulated kinase 1
SGK2	Serum and glucocorticoid regulated kinase 2
SGK3	Serum and glucocorticoid regulated kinase 3
SH2	Src homology 2
SHIP1	SH2 domain-containing inositol 5′-phosphatase 1
SHIP2	SH2 domain-containing inositol 5′-Phosphatase 2
SLC2A3	Solute carrier family 2, facilitated glucose transporter member 3
SLC43A1	Solute carrier family 43 member 1
SOS	Son of Sevenless
SPOP	Speckle type BTB/POZ protein
SRC-3	Steroid receptor co-activator 3
SRF	Serum response factor
SU2C-PCF IDT	Stand Up To Cancer & Prostate Cancer Foundation International Dream Team
T	Testosterone
TAK1	TGFβ-activated kinase 1
TAZ	Transcriptional coactivator with PDZ binding motif
TBC1D7	TBC1 Domain Family Member 7
TCF	T cell Factor
TCGA	The Cancer Genome Atlas
TEAD	Transcriptional enhanced associate domain
TEL2	Telomere length regulation protein
TGF	Transforming growth factor
TMPRSS2-ERG	Transmembrane protease, serine 2: ETS Transcription Factor fusion
TRAMP	Transgenic adenocarcinoma mouse prostate
TSC1	Tuberous Sclerosis complex 1
TSC2	Tuberous Sclerosis complex 2
TTI1	TELO2 interacting protein 1
Ub	Ubiquitination event
ULK1	Unc-51 Like Autophagy Activating Kinase 1
UVRAG	UV radiation resistance-associated gene; v-ATPase, Vacuolar (H+)-ATPase
VPS15	Vacuolar protein sorting 15
V-ATPase	Vacuolar H+-ATPase
WNT	WNT ligand
YAP	Yes-associated protein
