# Supplementary material for: The PI3K-AKT-mTOR Pathway and Prostate Cancer: At the Crossroads of AR, MAPK, and WNT Signaling
Source: Int J Mol Sci. 2020 Jun 25;21(12):4507. doi: 10.3390/ijms21124507 (PMC7350257; doi:10.3390/ijms21124507)
Supplement: Supplementary file 1 [file ijms-21-04507-s001.zip › Figure S1 revised.pptx]

## Slide 1
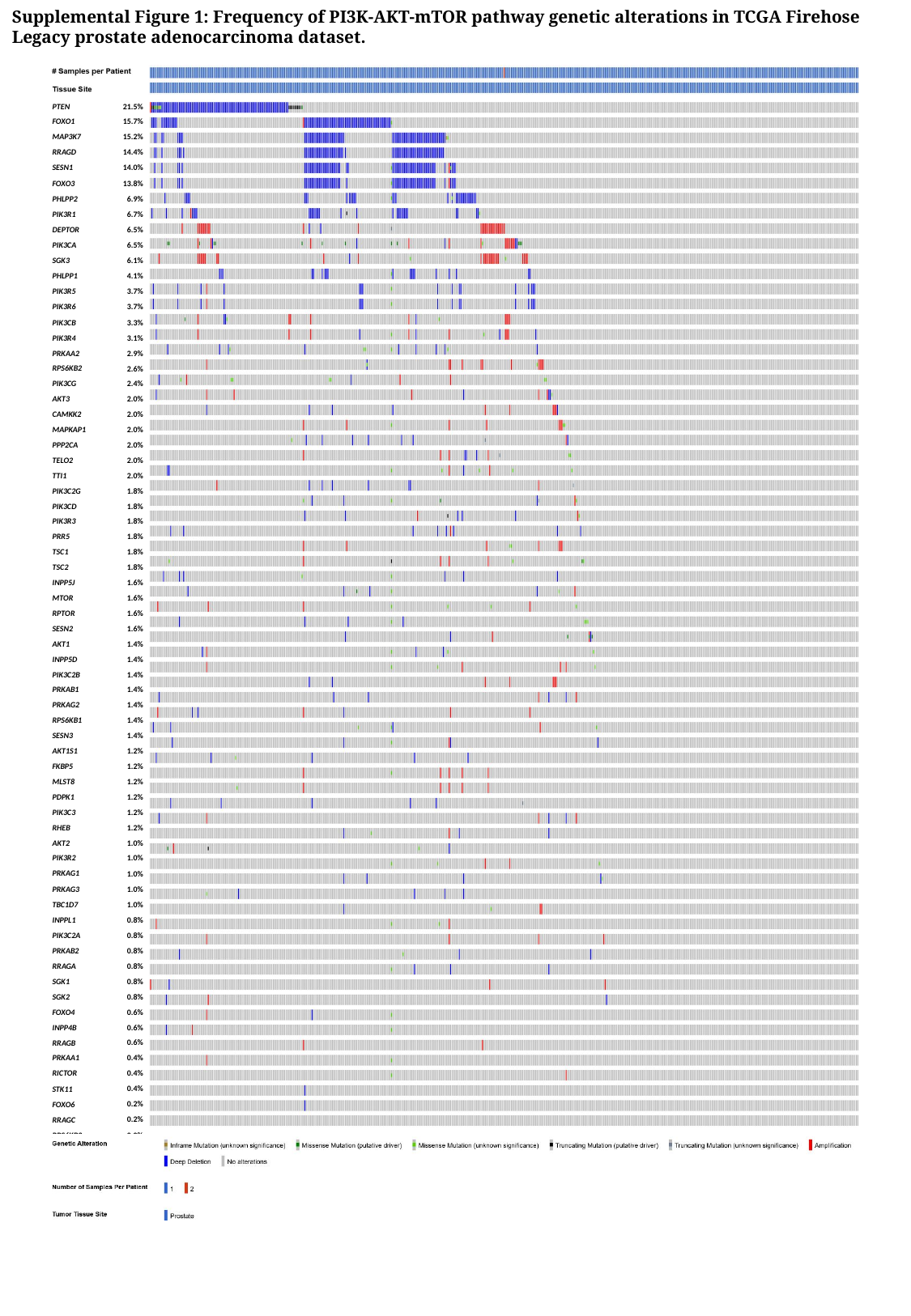

Supplemental Figure 1: Frequency of PI3K-AKT-mTOR pathway genetic alterations in TCGA Firehose Legacy prostate adenocarcinoma dataset.
PTEN
FOXO1
MAP3K7
RRAGD
SESN1
FOXO3
PHLPP2
PIK3R1
DEPTOR
PIK3CA
SGK3
PHLPP1
PIK3R5
PIK3R6
PIK3CB
PIK3R4
PRKAA2
RPS6KB2
PIK3CG
AKT3
CAMKK2
MAPKAP1
PPP2CA
TELO2
TTI1
PIK3C2G
PIK3CD
PIK3R3
PRR5
TSC1
TSC2
INPP5J
MTOR
RPTOR
SESN2
AKT1
INPP5D
PIK3C2B
PRKAB1
PRKAG2
RPS6KB1
SESN3
AKT1S1
FKBP5
MLST8
PDPK1
PIK3C3
RHEB
AKT2
PIK3R2
PRKAG1
PRKAG3
TBC1D7
INPPL1
PIK3C2A
PRKAB2
RRAGA
SGK1
SGK2
FOXO4
INPP4B
RRAGB
PRKAA1
RICTOR
STK11
FOXO6
RRAGC
RPS6KB3
21.5%
15.7%
15.2%
14.4%
14.0%
13.8%
6.9%
6.7%
6.5%
6.5%
6.1%
4.1%
3.7%
3.7%
3.3%
3.1%
2.9%
2.6%
2.4%
2.0%
2.0%
2.0%
2.0%
2.0%
2.0%
1.8%
1.8%
1.8%
1.8%
1.8%
1.8%
1.6%
1.6%
1.6%
1.6%
1.4%
1.4%
1.4%
1.4%
1.4%
1.4%
1.4%
1.2%
1.2%
1.2%
1.2%
1.2%
1.2%
1.0%
1.0%
1.0%
1.0%
1.0%
0.8%
0.8%
0.8%
0.8%
0.8%
0.8%
0.6%
0.6%
0.6%
0.4%
0.4%
0.4%
0.2%
0.2%
0.0%
