# Supplementary material for: The PI3K-AKT-mTOR Pathway and Prostate Cancer: At the Crossroads of AR, MAPK, and WNT Signaling
Source: Int J Mol Sci. 2020 Jun 25;21(12):4507. doi: 10.3390/ijms21124507 (PMC7350257; doi:10.3390/ijms21124507)
Supplement: Supplementary file 1 [file ijms-21-04507-s001.zip › Figure S2 revised.pptx]

## Slide 1
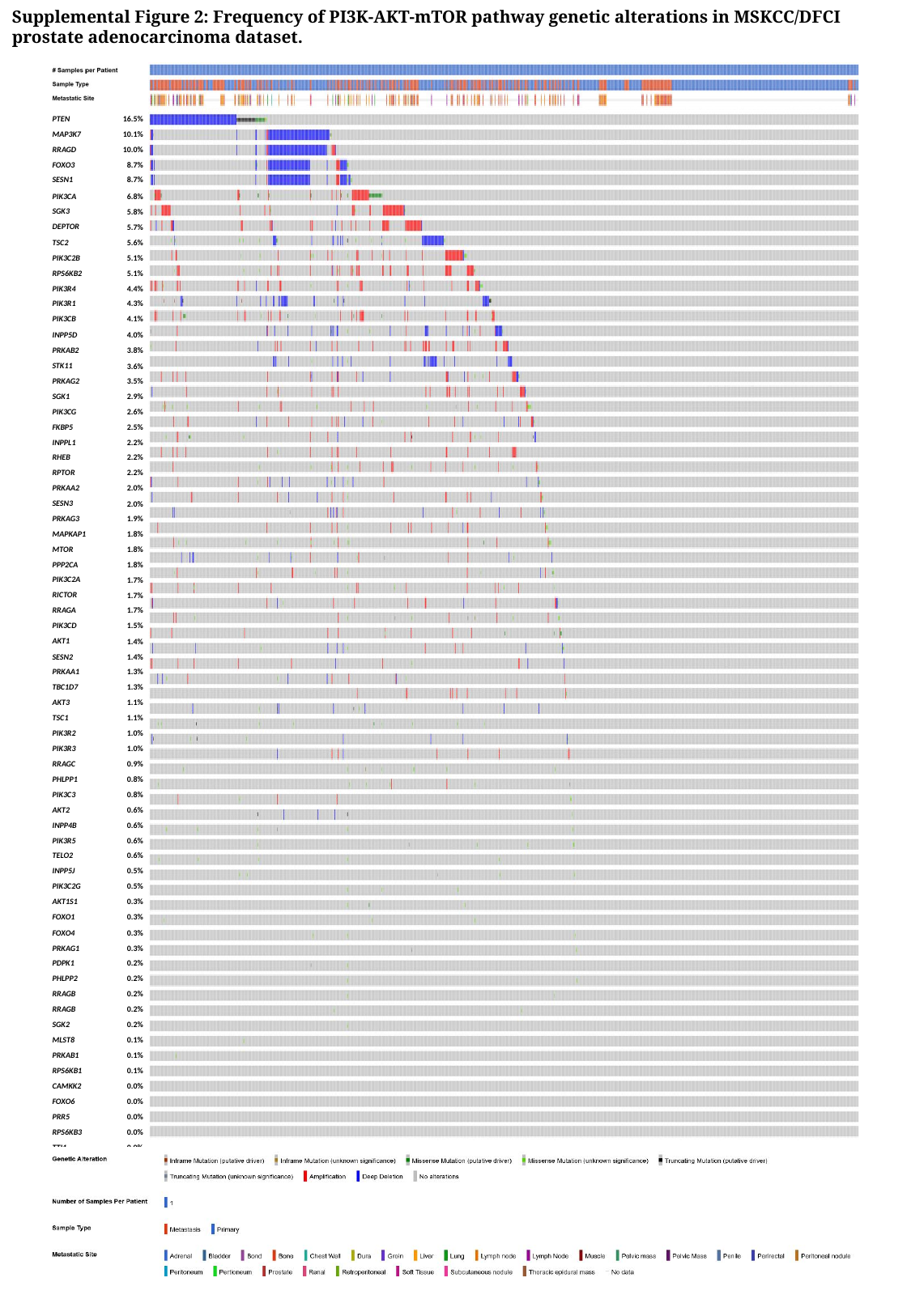

Supplemental Figure 2: Frequency of PI3K-AKT-mTOR pathway genetic alterations in MSKCC/DFCI prostate adenocarcinoma dataset.
PTEN
MAP3K7
RRAGD
FOXO3
SESN1
PIK3CA
SGK3
DEPTOR
TSC2
PIK3C2B
RPS6KB2
PIK3R4
PIK3R1
PIK3CB
INPP5D
PRKAB2
STK11
PRKAG2
SGK1
PIK3CG
FKBP5
INPPL1
RHEB
RPTOR
PRKAA2
SESN3
PRKAG3
MAPKAP1
MTOR
PPP2CA
PIK3C2A
RICTOR
RRAGA
PIK3CD
AKT1
SESN2
PRKAA1
TBC1D7
AKT3
TSC1
PIK3R2
PIK3R3
RRAGC
PHLPP1
PIK3C3
AKT2
INPP4B
PIK3R5
TELO2
INPP5J
PIK3C2G
AKT1S1
FOXO1
FOXO4
PRKAG1
PDPK1
PHLPP2
RRAGB
RRAGB
SGK2
MLST8
PRKAB1
RPS6KB1
CAMKK2
FOXO6
PRR5
RPS6KB3
TTI1
16.5%
10.1%
10.0%
8.7%
8.7%
6.8%
5.8%
5.7%
5.6%
5.1%
5.1%
4.4%
4.3%
4.1%
4.0%
3.8%
3.6%
3.5%
2.9%
2.6%
2.5%
2.2%
2.2%
2.2%
2.0%
2.0%
1.9%
1.8%
1.8%
1.8%
1.7%
1.7%
1.7%
1.5%
1.4%
1.4%
1.3%
1.3%
1.1%
1.1%
1.0%
1.0%
0.9%
0.8%
0.8%
0.6%
0.6%
0.6%
0.6%
0.5%
0.5%
0.3%
0.3%
0.3%
0.3%
0.2%
0.2%
0.2%
0.2%
0.2%
0.1%
0.1%
0.1%
0.0%
0.0%
0.0%
0.0%
0.0%
