# Supplementary material for: The PI3K-AKT-mTOR Pathway and Prostate Cancer: At the Crossroads of AR, MAPK, and WNT Signaling
Source: Int J Mol Sci. 2020 Jun 25;21(12):4507. doi: 10.3390/ijms21124507 (PMC7350257; doi:10.3390/ijms21124507)
Supplement: Supplementary file 1 [file ijms-21-04507-s001.zip › Figure S3 -revised.pptx]

## Slide 1
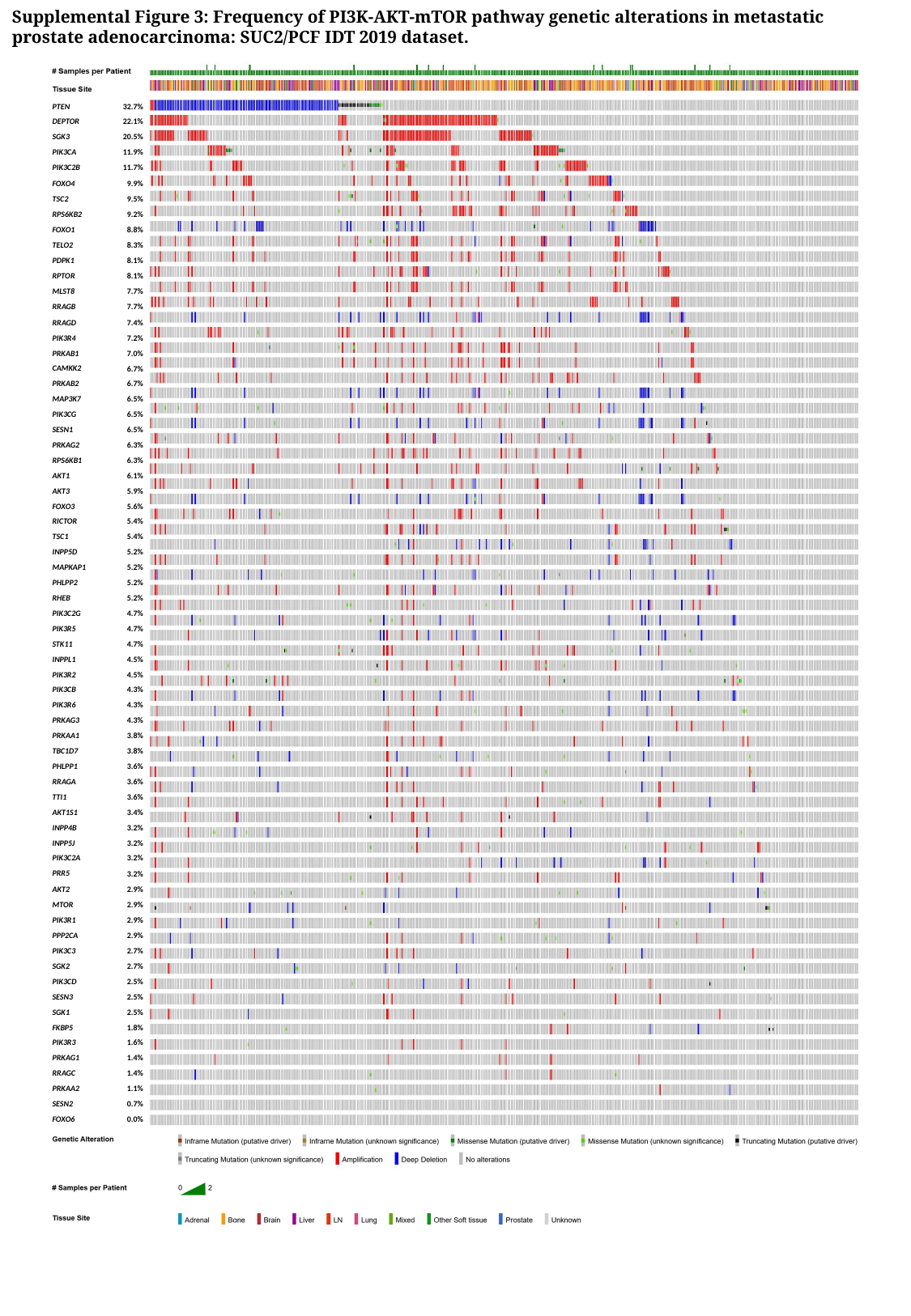

Supplemental Figure 3: Frequency of PI3K-AKT-mTOR pathway genetic alterations in metastatic prostate adenocarcinoma: SUC2/PCF IDT 2019 dataset.
PTEN
DEPTOR
SGK3
PIK3CA
PIK3C2B
FOXO4
TSC2
RPS6KB2
FOXO1
TELO2
PDPK1
RPTOR
MLST8
RRAGB
RRAGD
PIK3R4
PRKAB1
CAMKK2
PRKAB2
MAP3K7
PIK3CG
SESN1
PRKAG2
RPS6KB1
AKT1
AKT3
FOXO3
RICTOR
TSC1
INPP5D
MAPKAP1
PHLPP2
RHEB
PIK3C2G
PIK3R5
STK11
INPPL1
PIK3R2
PIK3CB
PIK3R6
PRKAG3
PRKAA1
TBC1D7
PHLPP1
RRAGA
TTI1
AKT1S1
INPP4B
INPP5J
PIK3C2A
PRR5
AKT2
MTOR
PIK3R1
PPP2CA
PIK3C3
SGK2
PIK3CD
SESN3
SGK1
FKBP5
PIK3R3
PRKAG1
RRAGC
PRKAA2
SESN2
FOXO6
RPS6KB3
32.7%
22.1%
20.5%
11.9%
11.7%
9.9%
9.5%
9.2%
8.8%
8.3%
8.1%
8.1%
7.7%
7.7%
7.4%
7.2%
7.0%
6.7%
6.7%
6.5%
6.5%
6.5%
6.3%
6.3%
6.1%
5.9%
5.6%
5.4%
5.4%
5.2%
5.2%
5.2%
5.2%
4.7%
4.7%
4.7%
4.5%
4.5%
4.3%
4.3%
4.3%
3.8%
3.8%
3.6%
3.6%
3.6%
3.4%
3.2%
3.2%
3.2%
3.2%
2.9%
2.9%
2.9%
2.9%
2.7%
2.7%
2.5%
2.5%
2.5%
1.8%
1.6%
1.4%
1.4%
1.1%
0.7%
0.0%
0.0%
