# Supplementary material for: The PI3K-AKT-mTOR Pathway and Prostate Cancer: At the Crossroads of AR, MAPK, and WNT Signaling
Source: Int J Mol Sci. 2020 Jun 25;21(12):4507. doi: 10.3390/ijms21124507 (PMC7350257; doi:10.3390/ijms21124507)
Supplement: Supplementary file 1 [file ijms-21-04507-s001.zip › Figure S4.pptx]

## Slide 1
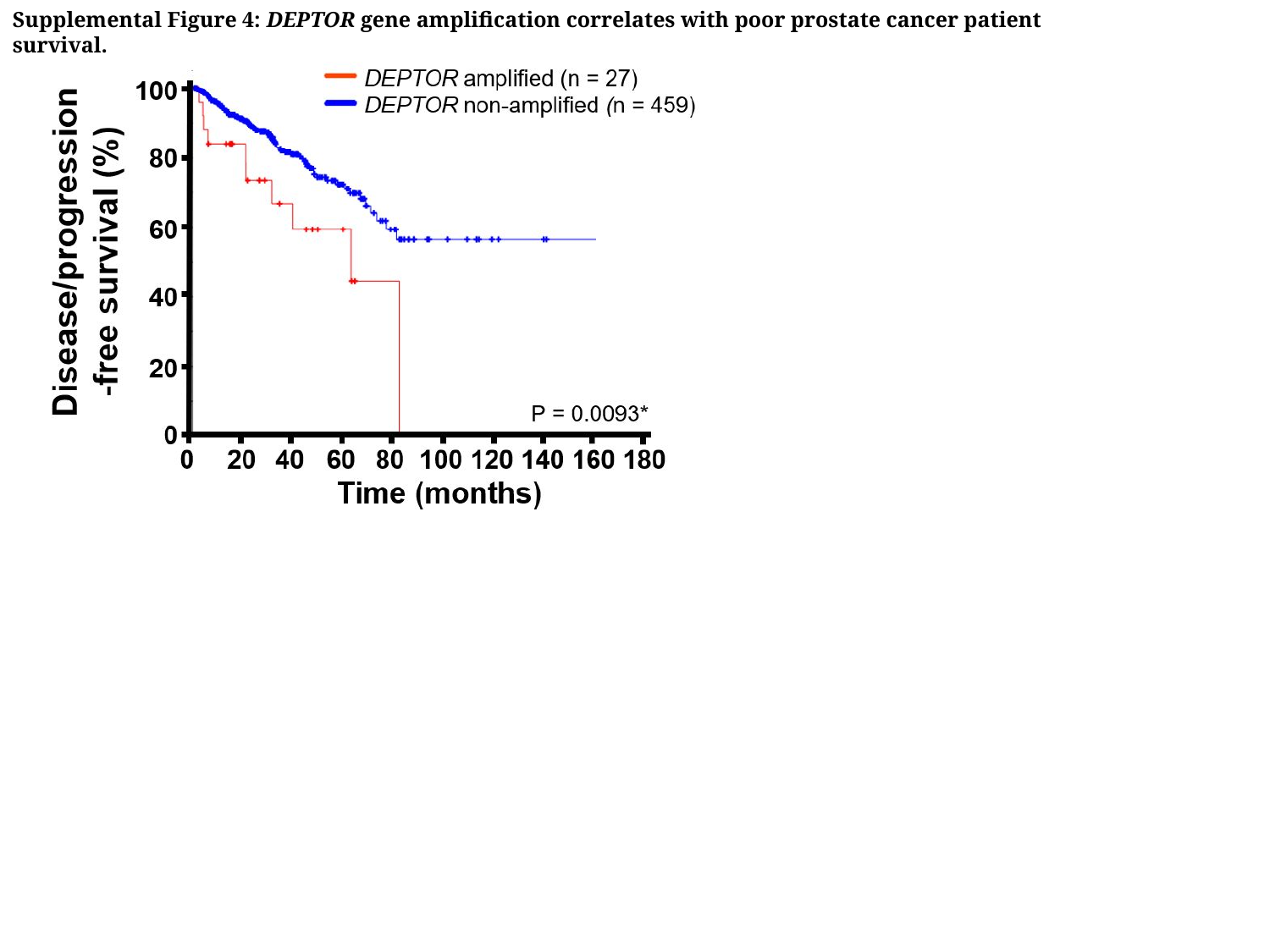

Supplemental Figure 4: DEPTOR gene amplification correlates with poor prostate cancer patient survival.
