# Supplementary material for: The PI3K-AKT-mTOR Pathway and Prostate Cancer: At the Crossroads of AR, MAPK, and WNT Signaling
Source: Int J Mol Sci. 2020 Jun 25;21(12):4507. doi: 10.3390/ijms21124507 (PMC7350257; doi:10.3390/ijms21124507)
Supplement: Supplementary file 1 [file ijms-21-04507-s001.zip › Suppl Table 2 Finalv2.docx]

| Table S2: Frequency of PI3K-AKT-mTOR pathway genetic alterations in prostate cancer: MSKCC/DFCI Prostate Adenocarcinoma dataset (n = 1,013, all samples with mutation and CNA data). | | | | | | |
| --- | --- | --- | --- | --- | --- | --- |
| Gene (protein) | | **Mutation**  **(%)** | **Amplification (%)** | **Deep deletion**  **(%)** | **Multiple alterations (%)** | **Total**  **(%)** |
| PI3K catalytic and regulatory subunits |  | | |  |  |  |
| Class IA | |  |  |  |  |  |
| *PIK3CA* (p110α) | | 2.76 | 3.95 | 0 | 0.1 | 6.81 |
| *PIK3CB* (p110β) | | 0.89 | 3.06 | 0 | 0.1 | 4.05 |
| *PIK3CD* (p110δ) | | 0.89 | 0.59 | 0 | 0 | 1.48 |
| *PIK3R1* (p85α) | | 0.99 | 0 | 3.36 | 0 | 4.34 |
| *PIK3R2* (p85β) | | 0.99 | 0 | 0 | 0 | 0.99 |
| *PIK3R3* (p55γ) | | 0.49 | 0 | 0.49 | 0 | 0.99 |
| Class IB | |  |  |  |  |  |
| *PIK3CG* (p110γ) | | 1.38 | 1.18 | 0 | 0 | 2.57 |
| *PIK3R5* (p101) | | 0.59 | 0 | 0 | 0 | 0.59 |
| *PIK3R6* (p87^PIKAP^/p84) | | 0.2 | 0 | 0 | 0 | 0.2 |
| Class II | |  |  |  |  |  |
| *PIK3C2A* (PI3KC2α) | | 0.79 | 0.79 | 0.1 | 0 | 1.68 |
| *PIK3C2B* (PI3KC2β) | | 0.99 | 3.95 | 0.2 | 0 | 5.13 |
| *PIK3C2G* (PI3KC2γ) | | 0.49 | 0 | 0 | 0 | 0.49 |
| Class III | |  |  |  |  |  |
| *PIK3C3* (VPS34) | | 0.59 | 0.2 | 0 | 0 | 0.79 |
| *PIK3R4* (p150, VPS15) | | 0.79 | 3.36 | 0.3 | 0 | 4.44 |
|  | |  |  |  |  |  |
| PI3K/AKT/mTOR signalling effector kinases | | | |  |  |  |
| *PDPK1* (PDK1) | | 0.2 | 0 | 0 | 0 | 0.2 |
| *AKT1* | | 0.39 | 0.79 | 0 | 0.2 | 1.38 |
| *AKT2* | | 0.3 | 0.3 | 0 | 0 | 0.59 |
| *AKT3* | | 0.1 | 0.99 | 0 | 0 | 1.09 |
| *RPS6KB1* (S6K1) | | 0.1 | 0 | 0 | 0 | 0.1 |
| *RPS6KB2* (S6K2) | | 0.69 | 4.34 | 0.1 | 0 | 5.13 |
| *RPS6KB3* (S6K3) | | 0 | 0 | 0 | 0 | 0 |
| *SGK1* | | 0.2 | 2.47 | 0.2 | 0 | 2.86 |
| *SGK2* | | 0.2 | 0 | 0 | 0 | 0.2 |
| *SGK3* | | 0.3 | 5.33 | 0.2 | 0 | 5.82 |
|  | |  |  |  |  |  |
| mTOR complex components | | |  |  |  |  |
| *AKT1S1* (PRAS40) | | 0.3 | 0 | 0 | 0 | 0.3 |
| *DEPTOR* | | 0 | 5.13 | 0.59 | 0 | 5.73 |
| *MTOR* | | 1.18 | 0.49 | 0 | 0.1 | 1.78 |
| *MLST8* (GβL) | | 0.1 | 0 | 0 | 0 | 0.1 |
| *MAPKAP1* (SIN1) | | 0.39 | 1.28 | 0.1 | 0 | 1.78 |
| *PRR5* (PROTOR) | | 0 | 0 | 0 | 0 | 0 |
| *RPTOR* (RAPTOR) | | 0.79 | 1.38 | 0 | 0 | 2.17 |
| *RICTOR* | | 0.39 | 1.18 | 0 | 0.1 | 1.68 |
| *TELO2* (TEL2) | | 0.59 | 0 | 0 | 0 | 0.59 |
| *TTI1* | | 0 | 0 | 0 | 0 | 0 |
| FOXO1 transcription factors | | |  |  |  |  |
| *FOXO1* | | 0.3 | 0 | 0 | 0 | 0.3 |
| *FOXO3* | | 0.3 | 0.79 | 7.6 | 0 | 8.69 |
| *FOXO4* | | 0.3 | 0 | 0 | 0 | 0.3 |
| *FOXO6* | | 0 | 0 | 0 | 0 | 0 |
|  | |  |  |  |  |  |
| PI3K/mTOR signalling regulation | | |  |  |  |  |
| *CAMKK2* (CaMKKβ) 0 | | | 0 | 0 | 0 | 0 |
| *MAP3K7* (TAK1) 0.39 | | | 0.3 | 9.38 | 0 | 10.07 |
| *PRKAA1* (AMPKα1) | | 0.1 | 0.89 | 0.3 | 0 | 1.28 |
| *PRKAA2* (AMPKα2) | | 0.49 | 0.49 | 0.99 | 0 | 1.97 |
| *PRKAB1* (AMPKβ1) | | 0.1 | 0 | 0 | 0 | 0.1 |
| *PRKAB2* (AMPKβ2) | | 0.1 | 3.36 | 0.3 | 0 | 3.75 |
| *PRKAG1* (AMPKγ1) | | 0.3 | 0 | 0 | 0 | 0.3 |
| *PRKAG2* (AMPKγ2) | | 0.49 | 2.07 | 0.89 | 0 | 3.46 |
| *PRKAG3* (AMPKγ3) | | 0.39 | 0.59 | 0.89 | 0 | 1.88 |
| *FKBP5* | | 0.2 | 1.58 | 0.69 | 0 | 2.47 |
| *INPP5D* (SHIP1) | | 0.59 | 0.59 | 2.76 | 0 | 3.95 |
| *INPPL1* (SHIP2) | | 0.89 | 0.99 | 0.3 | 0 | 2.17 |
| *INPP5J* (PIPP) | | 0.49 | 0 | 0 | 0 | 0.49 |
| *INPP4B* | | 0.3 | 0 | 0.3 | 0 | 0.59 |
| *PHLPP1* | | 0.79 | 0 | 0 | 0 | 0.79 |
| *PHLPP2* | | 0.2 | 0 | 0 | 0 | 0.2 |
| *PPP2CA* (PP2A) | | 0.49 | 0.39 | 0.89 | 0 | 1.78 |
| *PTEN* | | 4.24 | 0 | 12.14 | 0.1 | 16.49 |
| *TSC1* | | 0.3 | 0 | 0.69 | 0.1 | 1.09 |
| *TSC2* | | 1.28 | 0 | 4.24 | 0.1 | 5.63 |
| *TBC1D7* | | 0.3 | 0.49 | 0.49 | 0 | 1.28 |
| *STK11* (LKB1) | | 0.2 | 0 | 3.36 | 0 | 3.55 |
| *SESN1* | | 0.3 | 0.59 | 7.8 | 0 | 8.69 |
| *SESN2* | | 0.39 | 0.3 | 0.69 | 0 | 1.38 |
| *SESN3* | | 0.3 | 1.28 | 0.39 | 0 | 1.97 |
| *RHEB* | | 0.1 | 2.07 | 0 | 0 | 2.17 |
| *RRAGA* (RAGA) | | 0.2 | 0.99 | 0.49 | 0 | 1.68 |
| *RRAGB* (RAGB) | | 0.2 | 0 | 0 | 0 | 0.2 |
| *RRAGC* (RAGC) | | 0 | 0.69 | 0.2 | 0 | 0.89 |
| *RRAGD* (RAGD) | | 0 | 0.79 | 9.18 | 0 | 9.97 |
|  | |  |  |  |  |  |
