# Supplementary material for: The PI3K-AKT-mTOR Pathway and Prostate Cancer: At the Crossroads of AR, MAPK, and WNT Signaling
Source: Int J Mol Sci. 2020 Jun 25;21(12):4507. doi: 10.3390/ijms21124507 (PMC7350257; doi:10.3390/ijms21124507)
Supplement: Supplementary file 1 [file ijms-21-04507-s001.zip › Suppl Table 3 Finalv1.docx]

| Table S3: Frequency of PI3K-AKT-mTOR pathway genetic alterations in metastatic prostate adenocarcinoma; SUC2/PCF IDT dataset, PNAS 2019 (n = 444 samples, with mutation and CNA data). | | | | | | | | | | |
| --- | --- | --- | --- | --- | --- | --- | --- | --- | --- | --- |
| Gene (protein) | **Mutation**  **(%)** | | **Amplification**  **(%)** | | **Deep deletion**  **(%)** | | **Multiple alterations (%)** | | **Total**  **(%)** | |
| PI3K catalytic and regulatory subunits | |  | |  | |  | |  | |  |
| Class IA |  | |  | |  | |  | |  | |
| *PIK3CA* (p110α) | 2.7 | | 8.78 | | 0 | | 0.45 | | 11.94 | |
| *PIK3CB* (p110β) | 1.8 | | 2.25 | | 0 | | 0.23 | | 4.28 | |
| *PIK3CD* (p110δ) | 1.13 | | 0.45 | | 0.9 | | 0 | | 2.48 | |
| *PIK3R1* (p85α) | 1.58 | | 0.23 | | 1.13 | | 0 | | 2.93 | |
| *PIK3R2* (p85β) | 1.13 | | 2.93 | | 0.23 | | 0.23 | | 4.5 | |
| *PIK3R3* (p55γ) | 0.68 | | 0.45 | | 0.45 | | 0 | | 1.58 | |
| Class IB |  | |  | |  | |  | |  | |
| *PIK3CG* (p110γ) | 1.8 | | 3.6 | | 1.13 | | 0 | | 6.53 | |
| *PIK3R5* (p101) | 0.68 | | 1.13 | | 2.93 | | 0 | | 4.73 | |
| *PIK3R6* (p87^PIKAP^/p84) | 0 | | 1.35 | | 2.93 | | 0 | | 4.28 | |
| Class II |  | |  | |  | |  | |  | |
| *PIK3C2A* (PI3KC2α) | 1.13 | | 2.03 | | 0 | | 0 | | 3.15 | |
| *PIK3C2B* (PI3KC2β) | 1.35 | | 10.14 | | 0 | | 0.23 | | 11.71 | |
| *PIK3C2G* (PI3KC2γ) | 0.9 | | 2.93 | | 0.9 | | 0 | | 4.73 | |
| Class III |  | |  | |  | |  | |  | |
| *PIK3C3* (VPS34) | 0.9 | | 0.9 | | 0.9 | | 0 | | 2.7 | |
| *PIK3R4* (p150/VPS15) | 0.68 | | 6.53 | | 0 | | 0 | | 7.21 | |
|  |  | |  | |  | |  | |  | |
| PI3K/AKT/mTOR signalling effector kinases | |  | |  | |  | |  | |  |
| *PDPK1* (PDK1) | 0 | | 8.11 | | 0 | | 0 | | 8.11 | |
| *AKT1* | 0.9 | | 4.5 | | 0.68 | | 0 | | 6.08 | |
| *AKT2* | 0.45 | | 2.03 | | 0.45 | | 0 | | 2.93 | |
| *AKT3* | 0 | | 4.73 | | 1.13 | | 0 | | 5.86 | |
| *RPS6KB1* (S6K1) | 0 | | 6.31 | | 0 | | 0 | | 6.31 | |
| *RPS6KB2* (S6K2) | 0.68 | | 8.33 | | 0 | | 0.23 | | 9.23 | |
| *RPS6KB3* (S6K3) | 0 | | 0 | | 0 | | 0 | | 0 | |
| *SGK1* | 0.23 | | 2.03 | | 0.23 | | 0 | | 2.48 | |
| *SGK2* | 0 | | 2.03 | | 0.68 | | 0 | | 2.7 | |
| *SGK3* | 0.23 | | 20.27 | | 0 | | 0 | | 20.5 | |
|  |  | |  | |  | |  | |  | |
| mTOR complex components | | |  | |  | |  | |  | |
| *AKT1S1* (PRAS40) | 0.45 | | 2.7 | | 0.23 | | 0 | | 3.38 | |
| *DEPTOR* | 0.23 | | 21.4 | | 0 | | 0.45 | | 22.07 | |
| *MTOR* | 1.58 | | 0.23 | | 1.13 | | 0 | | 2.93 | |
| *MLST8* (GβL) | 0 | | 7.66 | | 0 | | 0 | | 7.66 | |
| *MAPKAP1* (SIN1) | 0.23 | | 4.5 | | 0.45 | | 0 | | 5.18 | |
| *PRR5* (PROTOR) | 0.23 | | 0.9 | | 2.03 | | 0 | | 3.15 | |
| *RPTOR* (RAPTOR) | 0.9 | | 6.98 | | 0.23 | | 0 | | 8.11 | |
| *RICTOR* | 0.23 | | 4.95 | | 0.23 | | 0 | | 5.41 | |
| *TELO2* (TEL2) | 0.68 | | 6.53 | | 0.9 | | 0.23 | | 8.33 | |
| *TTI1* | 0.23 | | 2.48 | | 0.9 | | 0 | | 3.6 | |
| FOXO1 transcription factors | | |  | |  | |  | |  | |
| *FOXO1* | 0.45 | | 0.23 | | 7.88 | | 0.23 | | 8.78 | |
| *FOXO3* | 0.23 | | 0.68 | | 4.5 | | 0.23 | | 5.63 | |
| *FOXO4* | 0.45 | | 8.78 | | 0.68 | | 0 | | 9.91 | |
| *FOXO6* | 0 | | 0 | | 0 | | 0 | | 0 | |
|  |  | |  | |  | |  | |  | |
| PI3K/mTOR signalling regulation | | |  | |  | |  | |  | |
| *CAMKK2* (CaMKKβ) 0 | | | 6.31 | | 0.45 | | 0 | | 6.67 | |
| *MAP3K7* (TAK1) 0.23 | | | 0.45 | | 5.86 | | 0 | | 6.53 | |
| *PRKAA1* (AMPKα1) | 0 | | 3.6 | | 0.23 | | 0 | | 3.83 | |
| *PRKAA2* (AMPKα2) | 0.45 | | 0.45 | | 0.23 | | 0 | | 1.13 | |
| *PRKAB1* (AMPKβ1) | 0.45 | | 6.31 | | 0.23 | | 0 | | 6.98 | |
| *PRKAB2* (AMPKβ2) | 0 | | 6.76 | | 0 | | 0 | | 6.76 | |
| *PRKAG1* (AMPKγ1) | 0.23 | | 1.13 | | 0 | | 0 | | 1.35 | |
| *PRKAG2* (AMPKγ2) | 0.9 | | 4.05 | | 1.35 | | 0 | | 6.31 | |
| *PRKAG3* (AMPKγ3) | 1.13 | | 2.03 | | 1.13 | | 0 | | 4.28 | |
| *FKBP5* | 0.23 | | 1.35 | | 0.23 | | 0 | | 1.8 | |
| *INPP5D* (SHIP1) | 0.68 | | 0.68 | | 3.83 | | 0 | | 5.18 | |
| *INPPL1* (SHIP2) | 1.13 | | 2.93 | | 0.23 | | 0.23 | | 4.5 | |
| *INPP5J* (PIPP) | 1.13 | | 0.9 | | 1.13 | | 0 | | 3.15 | |
| *INPP4B* | 0.45 | | 2.25 | | 0.45 | | 0 | | 3.15 | |
| *PHLPP1* | 1.13 | | 0.45 | | 2.03 | | 0 | | 3.6 | |
| *PHLPP2* | 1.13 | | 0.23 | | 3.83 | | 0 | | 5.18 | |
| *PPP2CA* (PP2A) | 0.68 | | 1.13 | | 1.13 | | 0 | | 2.93 | |
| *PTEN* | 6.31 | | 0.68 | | 25.68 | | 0 | | 32.66 | |
| *TSC1* | 0.68 | | 4.05 | | 0.68 | | 0 | | 5.41 | |
| *TSC2* | 1.8 | | 6.98 | | 0.68 | | 0 | | 9.46 | |
| *TBC1D7* | 0.23 | | 2.93 | | 0.68 | | 0 | | 3.83 | |
| *STK11* (LKB1) | 0.23 | | 1.58 | | 2.93 | | 0 | | 4.73 | |
| *SESN1* | 0.68 | | 1.13 | | 4.73 | | 0 | | 6.53 | |
| *SESN2* | 0.23 | | 0.23 | | 0.23 | | 0 | | 0.68 | |
| *SESN3* | 0.45 | | 1.58 | | 0.45 | | 0 | | 2.48 | |
| *RHEB* | 0 | | 4.05 | | 1.13 | | 0 | | 5.18 | |
| *RRAGA* (RAGA) | 0.68 | | 2.03 | | 0.9 | | 0 | | 3.6 | |
| *RRAGB* (RAGB) | 0 | | 7.66 | | 0 | | 0 | | 7.66 | |
| *RRAGC* (RAGC) | 0 | | 1.35 | | 0 | | 0 | | 1.35 | |
| *RRAGD* (RAGD) | 0 | | 0.9 | | 6.53 | | 0 | | 7.43 | |
|  |  | |  | |  | |  | |  | |
