# Supplementary material for: The PI3K-AKT-mTOR Pathway and Prostate Cancer: At the Crossroads of AR, MAPK, and WNT Signaling
Source: Int J Mol Sci. 2020 Jun 25;21(12):4507. doi: 10.3390/ijms21124507 (PMC7350257; doi:10.3390/ijms21124507)
Supplement: Supplementary file 1 [file ijms-21-04507-s001.zip › Supplemental material.docx]

**Supplemental material**

**Figure legends**

**Supplemental Figure 1: Frequency of PI3K-AKT-mTOR pathway genetic alterations in prostate cancer: TCGA Firehose Legacy prostate adenocarcinoma dataset.** OncoPrint displays the percentage frequency of genetic alterations in 68 genes that encode key components of the PI3K-AKT-mTOR signaling cascade in the TCGA firehose legacy prostate cancer dataset, n = 492 samples/patients (previously referred to as the Provisional TCGA prostate adenocarcinoma dataset in the literature). Only samples with mutation data from whole exome sequencing and putative copy number alteration (CNA) data (determined using GISTIC 2.0) were analysed. Data was accessed using the online cBioPortal cancer genomics platform [1-2].

**Supplemental Figure 2: Frequency of PI3K-AKT-mTOR pathway genetic alterations in prostate cancer: MSKCC/DFCI prostate adenocarcinoma dataset.** OncoPrint displays the percentage frequency of genetic alterations in 68 genes that encode key components of the PI3K-AKT-mTOR signaling cascade in the Memorial Sloan Kettering Cancer Center/Dana Farber Cancer Institute prostate adenocarcinoma dataset, n = 1,013 samples/patients [3]. Only samples with mutation data from whole exome sequencing and putative copy number alteration (CNA) data available were analysed. Data was accessed using the online cBioPortal cancer genomics platform [1-2].

**Supplemental Figure 3: Frequency of PI3K-AKT-mTOR pathway genetic alterations in metastatic prostate adenocarcinoma: SUC2/PCF IDT 2019 dataset.** OncoPrint displays the percentage frequency of genetic alterations in 68 genes that encode key components of the PI3K-AKT-mTOR signaling cascade in the Stand Up To Cancer & Prostate Cancer Foundation International Dream Team (SU2C-PCF IDT) dataset, n = 444 samples (collected from 429 patients) [4]. Only samples with mutation data from whole exome sequencing and putative copy number alteration (CNA) data available were analysed. Data was accessed using the online cBioPortal cancer genomics platform [1-2].

**Supplemental Figure 4: *DEPTOR* gene amplification correlates with poor prostate cancer patient survival.** Disease/progression-free Kaplan-Meier estimate plot for *DEPTOR* gene amplification carriers relative to *DEPTOR* unamplified patients within the TCGA Firehose legacy prostate adenocarcinoma patient genomic dataset (n = 492). *Log-rank test p-value = 0.0093. Data accessed using the cBioPortal [1-2].

**Table legends**

**Supplemental Table 1: Frequency of PI3K-AKT-mTOR pathway genetic alterations in prostate cancer: TCGA Firehose Legacy prostate adenocarcinoma dataset.** Table displays the percentage frequency of genetic alterations in 68 genes that encode key components of the PI3K-AKT-mTOR signaling cascade in the TCGA firehose legacy prostate cancer dataset, n = 492 samples/patients (previously referred to as the Provisional TCGA prostate adenocarcinoma dataset in the literature). Only samples with mutation data from whole exome sequencing and putative copy number alteration (CNA) data (determined using GISTIC 2.0) were analysed, and the percentage frequency was rounded to 2 decimal places. Deletion and amplification columns represent putative copy-number calls for homozygous deletion (value = -2) and high-level amplification (value = 2) respectively. Data was accessed using the online cBioPortal cancer genomics platform [1-2].

**Supplemental Table 2: Frequency of PI3K-AKT-mTOR pathway genetic alterations in prostate cancer: MSKCC/DFCI prostate adenocarcinoma dataset.** Table displays the percentage frequency of genetic alterations in 68 genes that encode key components of the PI3K-AKT-mTOR signaling cascade in the Memorial Sloan Kettering Cancer Center/Dana Farber Cancer Institute prostate adenocarcinoma dataset, n = 1,013 samples/patients [3]. Only samples with mutation data from whole exome sequencing and putative copy number alteration (CNA) data available were analysed, and the percentage frequency rounded to 2 decimal places. Deletion and amplification columns represent putative copy-number calls for homozygous deletion (value = -2) and high-level amplification (value = 2) respectively. Data was accessed using the online cBioPortal cancer genomics platform [1-2].

**Supplemental Table 3: Frequency of PI3K-AKT-mTOR pathway genetic alterations in metastatic prostate adenocarcinoma: SUC2/PCF IDT 2019 dataset.** Table displays the percentage frequency of genetic alterations in 68 genes that encode key components of the PI3K-AKT-mTOR signaling cascade in the Stand Up To Cancer & Prostate Cancer Foundation International Dream Team (SU2C-PCF IDT) dataset, n = 444 samples (collected from 429 patients) [4]. Only samples with mutation data from whole exome sequencing and putative copy number alteration (CNA) data available were analysed, and the percentage frequency rounded to 2 decimal places. Deletion and amplification columns represent putative copy-number calls for homozygous deletion (value = -2) and high-level amplification (value = 2) respectively. Data was accessed using the online cBioPortal cancer genomics platform [1-2].

**References:**

1. Cerami, E.; Gao, J.; Dogrusoz, U.; Gross, B. E.; Sumer, S. O.; Aksoy, B. A.; Jacobsen, A.; Byrne, C. J.; Heuer, M. L.; Larsson, E.; Antipin, Y.; Reva, B.; Goldberg, A. P.; Sander, C.; Schultz, N., The cBio cancer genomics portal: an open platform for exploring multidimensional cancer genomics data. *Cancer Discov* **2012,** *2* (5), 401-4. DOI: 10.1158/2159-8290.cd-12-0095

2. Gao, J.; Aksoy, B. A.; Dogrusoz, U.; Dresdner, G.; Gross, B.; Sumer, S. O.; Sun, Y.; Jacobsen, A.; Sinha, R.; Larsson, E.; Cerami, E.; Sander, C.; Schultz, N., Integrative analysis of complex cancer genomics and clinical profiles using the cBioPortal. *Science signaling* **2013,** *6* (269), pl1. DOI: 10.1126/scisignal.2004088

3. Armenia, J.; Wankowicz, S. A. M.; Liu, D.; Gao, J.; Kundra, R.; Reznik, E.; Chatila, W. K.; Chakravarty, D.; Han, G. C.; Coleman, I.; Montgomery, B.; Pritchard, C.; Morrissey, C.; Barbieri, C. E.; Beltran, H.; Sboner, A.; Zafeiriou, Z.; Miranda, S.; Bielski, C. M.; Penson, A. V.; Tolonen, C.; Huang, F. W.; Robinson, D.; Wu, Y. M.; Lonigro, R.; Garraway, L. A.; Demichelis, F.; Kantoff, P. W.; Taplin, M. E.; Abida, W.; Taylor, B. S.; Scher, H. I.; Nelson, P. S.; de Bono, J. S.; Rubin, M. A.; Sawyers, C. L.; Chinnaiyan, A. M.; Schultz, N.; Van Allen, E. M., The long tail of oncogenic drivers in prostate cancer. *Nat Genet* **2018,** *50* (5), 645-651. DOI: 10.1038/s41588-018-0078-z

4. Abida, W.; Cyrta, J.; Heller, G.; Prandi, D.; Armenia, J.; Coleman, I.; Cieslik, M.; Benelli, M.; Robinson, D.; Van Allen, E. M.; Sboner, A.; Fedrizzi, T.; Mosquera, J. M.; Robinson, B. D.; De Sarkar, N.; Kunju, L. P.; Tomlins, S.; Wu, Y. M.; Nava Rodrigues, D.; Loda, M.; Gopalan, A.; Reuter, V. E.; Pritchard, C. C.; Mateo, J.; Bianchini, D.; Miranda, S.; Carreira, S.; Rescigno, P.; Filipenko, J.; Vinson, J.; Montgomery, R. B.; Beltran, H.; Heath, E. I.; Scher, H. I.; Kantoff, P. W.; Taplin, M.-E.; Schultz, N.; deBono, J. S.; Demichelis, F.; Nelson, P. S.; Rubin, M. A.; Chinnaiyan, A. M.; Sawyers, C. L., Genomic correlates of clinical outcome in advanced prostate cancer. *Proceedings of the National Academy of Sciences* **2019,** *116* (23), 11428. DOI: 10.1073/pnas.1902651116
